# Single-cell landscape of melanoma reveals ETV5-driven C3 ID4^+^ tumor subpopulation with extracellular vesicle-associated immunosuppressive and pro-metastatic potential

**DOI:** 10.3389/fimmu.2026.1795778

**Published:** 2026-04-16

**Authors:** Haibo Li, Xin Zheng, Yue Gong, Mengran Xi, Haoyu Sun, Yantao Ding, Qi Sun

**Affiliations:** 1 Department of Plastic and Reconstructive Surgery, Shanghai Ninth People’s Hospital, Shanghai Jiao Tong University School of Medicine, Shanghai, China; 2 Department of Facial Plastic and Reconstructive Surgery, Eye & ENT Hospital of Fudan University, Shanghai, China; 3 School of Medicine, Shanghai Jiao Tong University, Shanghai, China; 4 Department of Dermatology, Shanghai Minhang District Traditional Chinese Medicine Hospital, Shanghai, China; 5 Fudan University–Doctor Kong Joint Research Center for Sports Medicine and Health Footwear, Fudan University Institute of Sports Medicine, Jinqiao Laboratory, Shanghai, China; 6 Department of Orthopaedic Surgery, China-Japan Union Hospital of Jilin University, Changchun, China; 7 Department of Dermatology, The First Affiliated Hospital, Anhui Medical University, Hefei, Anhui, China; 8 Key Laboratory of Dermatology, Anhui Medical University, Ministry of Education, Hefei, Anhui, China; 9 Department of Radiology, Shanghai Ninth People’s Hospital, Shanghai Jiao Tong University School of Medicine, Shanghai, China

**Keywords:** ETV5, extracellular vesicles, malignant melanoma, single-cell RNA sequencing, TGF-β signaling pathway, tumor microenvironment

## Abstract

**Background:**

Malignant melanoma is characterized by marked intratumoral heterogeneity and an immunosuppressive tumor microenvironment (TME), and resistance to immunotherapy remains common. We hypothesized that melanoma contains a poorly differentiated tumor subpopulation characterized by an ETV5-centered transcriptional program and TGF-β-associated intercellular crosstalk, which may contribute to malignant progression and immune evasion. EV-related signaling was explored as a potential, but unvalidated, component of this phenotype.

**Methods:**

We performed integrative single-cell transcriptomic analyses of single-cell RNA sequencing (scRNA-seq) data from 10 Stage I/III melanoma specimens to deconvolute the tumor microenvironment and tumor heterogeneity. Analyses included clustering, differential expression, stemness prediction (CytoTRACE), pseudotime trajectory reconstruction, cell-cell communication (CellChat), and transcriptional regulatory network inference (SCENIC). Functional validation was performed using CRISPR/Cas9-mediated ETV5 knockdown, followed by migration, proliferation, apoptosis, and RT-qPCR assays.

**Results:**

We identified 10 major cell types and 6 distinct tumor subpopulations. The C3 ID4^+^ tumor cell (TC) subpopulation was markedly enriched in Stage III tumors and showed a high-risk phenotype. It was characterized by enhanced proliferation, oxidative phosphorylation, stemness, and impaired immune activation. This subpopulation also expressed high levels of ID4, SPP1, and POSTN. Pseudotime analysis placed C3 ID4^+^ TCs at a poorly differentiated state along the developmental trajectory. Cell-cell communication analysis revealed extensive crosstalk between C3 ID4^+^ TCs and C0 GMPR^+^ TCs through the TGFB2-(TGFBR1+TGFBR2) signaling axis. ETV5 was further identified as a key transcriptional regulator in this subpopulation. Functional validation showed that ETV5 knockdown reduced melanoma-cell migration and proliferation and promoted apoptosis.

**Conclusion:**

Our integrative single-cell analysis identifies C3 ID4^+^ TCs as a critical driver of melanoma progression, associated with metabolic reprogramming, immune evasion, and TGF-β-related intercellular crosstalk. We further demonstrate that the transcriptional regulator ETV5 is functionally required to maintain this aggressive phenotype. These findings nominate ETV5 and TGF-β-associated signaling as candidate therapeutic vulnerabilities in melanoma enriched for this high-risk subpopulation.

## Introduction

Malignant melanoma is a highly aggressive skin tumor originating from melanocytes, with its incidence continuing to rise globally, particularly among Caucasian populations (1–3). Although early-stage melanoma can often be effectively treated with surgical excision, the prognosis deteriorates significantly once the disease progresses to advanced stages—especially with lymph node involvement or distant metastasis (Stages III and IV)—leading to a markedly reduced five-year survival rate (3–7). In recent years, tumor immunosuppressants and therapeutic markers have been developed and studied in various types of cancer (8, 9). In the case of malignant melanoma, the application of immune checkpoint inhibitors (such as anti-PD-1 and anti-CTLA-4 antibodies) and targeted therapies (such as BRAF/MEK inhibitors) has significantly improved the survival outcomes of some patients in advanced stages. However, due to development limitations, it still faces many challenges, including treatment response heterogeneity, drug resistance, and immune-related adverse events (10–13). In metastatic melanoma, baseline FT3, A-TPO, LDH, and M stage have been reported to independently predict overall survival after anti–PD-1 therapy and can be integrated into a prognostic nomogram (14). Therefore, the advancement of melanoma treatment requires high-resolution methods, as well as the construction of prognostic models and personalized treatment plans as mentioned in other studies (15), which can address the heterogeneity of tumors and their microenvironments, and identify actionable progression drivers and treatment resistance. Single-cell transcriptomic analysis provides a particularly useful framework for moving beyond descriptive feature descriptions and towards identifying key cellular states and vulnerabilities in a mechanism-oriented manner.

TME is a complex ecosystem composed of diverse cell types, their secreted factors, extracellular matrix, and other components (16), playing a critical role in tumor initiation, progression, immune evasion, and therapy resistance (13, 17–20). In melanoma, the TME encompasses not only the tumor cells themselves but also infiltrating immune cells (such as T cells, natural killer cells, macrophages, and dendritic cells), stromal cells (including fibroblasts, endothelial cells, and pericytes), and non-cellular components (such as cytokines and chemokines) (21, 22). These components interact through an intricate intercellular communication network, collectively shaping the immune phenotype and malignant behavior of the tumor. Research has shown that interactions between melanoma cells and immune cells within the TME—particularly those mediated by signaling pathways such as TGF-β and PD-1/PD-L1—significantly influence the efficacy of anti-tumor immune responses (23–26). For instance, ectopic expression of GDF15 in cancer-associated fibroblasts has been shown to significantly enhance immunosuppression in melanoma (27). Heterogeneity in melanoma is not only observed among tumor cells but is also widely present across immune and stromal cell subpopulations within the TME. This heterogeneity is a major contributor to the considerable individual variation in treatment responses. For instance, tumor-associated macrophages (TAMs) in melanoma can polarize into a pro-tumoral M2 phenotype, which suppresses T cell function and promotes angiogenesis. Dendritic cells (DCs), on the other hand, play a key role in antigen presentation and T cell activation, though their function is often suppressed in the tumor microenvironment (28–32). Furthermore, melanoma cells themselves exhibit significant transcriptional heterogeneity, with distinct subpopulations showing marked differences in cell cycle regulation, metabolic reprogramming, and stemness characteristics—features closely associated with metastatic potential and therapy resistance (33, 34).

EVs have emerged as pivotal mediators of intercellular communication within the TME, capable of transferring proteins, lipids, and nucleic acids to modulate immune responses, metastasis, and therapy resistance (35–37). In melanoma, tumor-derived EVs have been shown to carry immunosuppressive cargo (e.g., PD-L1, TGF-β) and promote pre-metastatic niche formation (38, 39). However, the specific tumor subpopulations that serve as major sources of these pathogenic EVs, and the transcriptional programs governing their EV-related secretory phenotype, remain largely unknown.

In recent years, the rapid advancement of single-cell RNA sequencing (scRNA-seq) technology has provided unprecedented resolution for systematically dissecting the cellular composition, state transitions, and intercellular communication within the tumor microenvironment (40–43). Using scRNA-seq, researchers can identify novel cell subpopulations, uncover trajectories of cellular state transitions, and pinpoint key driver genes and signaling pathways at single-cell resolution, thereby offering deeper insights into tumor biology. In melanoma research, several studies have leveraged this technology to reveal the diversity of intratumoral cell subsets, immune infiltration profiles, and their associations with clinical outcomes (44–48). However, many questions remain unanswered regarding the dynamic evolution of the TME between early and advanced stages (such as Stage I and Stage III), the characteristics of key tumor subpopulations, and their roles in disease progression.

## Methods

### Acquisition of single-cell data

The single-cell RNA sequencing data utilized in this study were obtained from the GEO database (https://www.ncbi.nlm.nih.gov/geo/) under accession number GSE277165. Using the Seurat package (v4.3.0), we loaded the 10x Genomics data for each sample into R (v4.3.3). An initial quality control step was performed with DoubletFinder (v2.0.3) to filter out potential doublets and low-quality cells. Cells were retained for downstream analysis only if they met the following criteria: number of detected genes (nFeature_RNA) between 500 and 6000, and mitochondrial gene content below 25% of total transcripts.

### Differential gene characterization and enrichment analysis

Differentially expressed genes (DEGs) for each cell cluster and subpopulation were identified using the “FindAllMarkers” function with Wilcoxon rank-sum test as the default method (log_2_FC threshold > 0.25). To elucidate the functional roles of these DEGs, enrichment analysis was performed using the clusterProfiler (v4.6.2) and SCP (v0.4.8) packages. Significantly enriched pathways for each cell cluster and subpopulation were derived from Gene Ontology (GO) analysis (49). Additionally, the AUCell algorithm was applied to identify active gene sets and transcription factor activities in the scRNA-seq data. No dedicated extracellular-vesicle gene set was used for enrichment scoring in the current study; EV-related interpretations were based on literature-informed evaluation of individual EV-associated genes rather than on a predefined EV signature. This EV-related interpretation was not derived from a formal EV-specific enrichment analysis and should therefore be interpreted as hypothesis-generating.

### Pseudotime trajectory construction

Using the Monocle2 package (v2.24.0), we reconstructed pseudotemporal trajectories of malignant melanoma tumor cell subpopulations based on scRNA-seq data. This approach enables the characterization of dynamic cellular transitions and state changes occurring during tumor cell differentiation in melanoma.

### CytoTRACE and slingshot analysis

To investigate developmental and differentiation states among malignant melanoma tumor cell subpopulations, we applied CytoTRACE to assess and rank differentiation states across all tumor cells. Additionally, using the Slingshot package (v2.6.0), we inferred developmental trajectories and characterized dynamic gene expression changes along pseudotime (50). The “getLineages” and “getCurves” functions were employed to reconstruct branching trajectories and evaluate temporal expression patterns of differentially expressed genes during tumor progression.

### Analysis of cellular crosstalk

Intercellular communication was systematically investigated using the CellChat computational framework (v1.6.1) to infer cell-cell signaling networks from single-cell transcriptomic profiles (2, 51). The analysis encompassed all identified cell populations, including distinct tumor subpopulations and their microenvironmental counterparts. The CellChatDB.human database provided the foundational ligand-receptor interaction repository for these predictions, with statistical significance determined at P < 0.05. Our investigation placed particular emphasis on delineating the communication axes between pivotal tumor subpopulations, notably the signaling pathways operating between C0 GMPR^+^ TCs and C3 ID4^+^ TCs. Through sophisticated visualization approaches, we aimed to elucidate the key molecular pathways that potentially govern the functional crosstalk between these malignant subsets. CellChatDB.human primarily captures ligand-receptor interactions mediated by secreted or membrane-associated molecules and does not explicitly model vesicle-mediated signal transfer; no dedicated EV-specific interaction resource, such as ExoCarta, was incorporated into the current analysis.

### SCENIC analysis for gene regulatory network reconstruction

Gene regulatory networks were reconstructed from scRNA-seq data using SCENIC (Single-Cell Regulatory Network Inference and Clustering) to identify stable cellular states. The analysis was performed using the pySCENIC module (v0.12.1) in Python (v3.9.19), with subsequent visualization and downstream analysis conducted in R (v4.3.3). This workflow included generation of AUCell matrices to quantitatively assess transcription factor enrichment and evaluate regulon activity across cell populations.

### Cell culture

Human melanoma cell lines A375 and MEWo were obtained from the Cell Bank of the Chinese Academy of Sciences (Shanghai, China) and authenticated by STR profiling. Cells were cultured in Dulbecco’s Modified Eagle Medium (DMEM, Gibco, Cat#11965092) supplemented with 10% fetal bovine serum (FBS, Gibco, Cat#10099141C) and 1% penicillin–streptomycin (Gibco, Cat#15140122) at 37 °C in a humidified atmosphere with 5% CO_2_. All cell lines were routinely tested negative for mycoplasma contamination.

### Lentiviral sgRNA construction and transduction

To knock down ETV5, two independent sgRNAs targeting coding exons conserved across major transcripts (RefSeq NM_004454.5) were designed and cloned into lentiCRISPR v2 (Addgene #52961) following the Zhang lab protocol. Oligonucleotides were synthesized with BsmBI-compatible overhangs and annealed before ligation (52).

sg-ETV5#1 (protospacer, sense strand, 5’→3’):

GAGCGGATGACCTGCTACGA (PAM: TGG).

Cloning oligos:

- Forward: CACCGAGCGGATGACCTGCTACGA.
- Reverse: AAACTCGTAGCAGGTCATCCGCTC.

sg-ETV5#2 (protospacer, sense strand, 5’→3’):

CGGCTGCTTCTACACCGTGA (PAM: AGG).

Cloning oligos:

- Forward: CACCGCGGCTGCTTCTACACCGTGA.
- Reverse: AAACTCACGGTGTAGAAGCAGCCG.

Non-targeting control (sg-Ctrl) protospacer:

GCGAGGTATTCGGCTCCGCG (PAM: TGG).

Cloning oligos:

- Forward: CACCGGCGAGGTATTCGGCTCCGCG.
- Reverse: AAACCGCGGAGCCGAATACCTCGC.

Lentiviral particles were packaged in HEK293T cells using psPAX2 (Addgene #12260) and pMD2.G (Addgene #12259) with Lipofectamine 3000 (Invitrogen, Cat#L3000015). Supernatants were collected at 48 h and 72 h, filtered (0.45 μm), optionally concentrated (Lenti-X Concentrator, Takara, Cat#631231), and used to infect A375 and MEWo cells in the presence of 8 μg/mL polybrene (Sigma, Cat#H9268). Stable pools were selected with 2 μg/mL puromycin (Sigma, Cat#P8833) for 72 h and maintained in 1 μg/mL thereafter. Editing was verified by genomic PCR–Sanger around the target site and by qRT-PCR at the mRNA level. Use human genome build GRCh38 for design/validation; confirm absence of common SNPs within protospacers; keep MOI ≤0.3 for single-copy editing if making clonal lines.

### Quantitative real-time PCR

Total RNA was extracted using TRIzol (Invitrogen, Cat#15596026). cDNA was synthesized with PrimeScript RT Reagent Kit (Takara, Cat#RR037A) including gDNA Eraser. qPCR was performed using TB Green Premix Ex Taq II (Takara, Cat#RR820A) on a Bio-Rad CFX96. Each reaction (10 μL) contained 1× TB Green mix, 0.4 μM primers, and ~10 ng cDNA. Cycling: 95 °C 30 s, then 40 cycles of 95 °C 5 s/60 °C 30 s; followed by melt-curve analysis. GAPDH served as the internal reference; relative expression was calculated by 2^-ΔΔCt.

ETV5 qPCR primers (span exon–exon junction; amplicon ~132 bp):

- Forward (5’→3’): AGCAGCAGATGAAGACCAGC.
- Reverse (5’→3’): CTGTTGTTGGTGCTGGTGTT.

GAPDH qPCR primers (amplicon ~142 bp):

- Forward (5’→3’): GGAGCGAGATCCCTCCAAAAT.
- Reverse (5’→3’): GGCTGTTGTCATACTTCTCATGG.

Each sample was run in technical triplicate across ≥3 biological replicates. Primer specificity and single-peak melt curves were confirmed prior to formal runs; amplification efficiency (90–110%) was verified using a 5-point 10-fold dilution series (53).

### Wound healing assay

Cells were seeded into 6-well plates and grown to ~90% confluence. A uniform scratch was generated using a 200 μL sterile pipette tip, and floating cells were removed with PBS. Cells were then maintained in serum-free DMEM for 72 h. Images were captured at 0 h and 72 h using an inverted microscope (Olympus IX71), and wound closure was quantified with ImageJ.

### Transwell migration assay

Cell migration was examined using 24-well Transwell chambers with 8-μm pores (Corning, Cat#3422). A total of 5 × 10^4^ cells in serum-free medium were seeded into the upper chamber, while the lower chamber contained 10% FBS as chemoattractant. After 24 h, migrated cells were fixed with 4% paraformaldehyde and stained with 0.1% crystal violet, followed by imaging and quantification in five random fields.

### Colony formation assay

500–800 cells were seeded per well in 6-well plates and cultured for 10–14 days. Colonies were fixed with methanol and stained with crystal violet. Colonies containing >50 cells were counted manually.

### Cell proliferation assay

Cells were plated into 96-well plates (2 × 10³ cells/well) and monitored for 4 days using the Cell Counting Kit-8 (Dojindo, Cat#CK04). Absorbance was measured at 450 nm using a BioTek Synergy HTX reader.

### Apoptosis assay by flow cytometry

Cell apoptosis was assessed using the Annexin V-FITC/PI Apoptosis Detection Kit (BD Biosciences, Cat#556547). Stained cells were analyzed with a BD FACSCanto II cytometer, and apoptotic fractions were quantified using FlowJo (v10).

### Statistical analysis

Data are presented as mean ± SD from at least three independent experiments. Statistical significance was determined using Student’s t-test or one-way ANOVA with Tukey’s *post hoc* test in GraphPad Prism 9. A value of p < 0.05 was considered statistically significant.

## Results

### Heterogeneity of the tumor microenvironment in malignant melanoma

We selected 10 malignant melanoma tissue samples at stages I and III from a public database. Following rigorous quality control and batch effect removal, 42,317 high-quality cells were obtained. Through dimensionality reduction and clustering analysis, we identified 10 distinct cell clusters. Based on differential expression of established cell-type-specific markers, these clusters were annotated as follows: melanoma cells, fibroblasts, T/NK cells, macrophages, endothelial cells (ECs), keratinocytes, pericytes, B/plasma cells, mast cells (MCs), and dendritic cells (DCs) (**Supplementary Figure 1A**). The top five marker genes for each cell type are displayed in the bubble plot in **Supplementary Figure 1B**, with ECT, PMEL, MIA, S100A1, and S100B identified as the top markers for melanoma cells. Melanoma cells were notably more abundant in stage III tumors and were predominantly in the G2/M and S phases of the cell cycle (**Supplementary Figures 1C–E**). Moreover, melanoma cells exhibited the highest nCount RNA values, followed by DCs and macrophages (**Supplementary Figure 1F**). In terms of stemness, ECs, pericytes, and melanoma cells showed the highest scores, while fibroblasts also displayed considerable stemness, though to a lesser extent (**Supplementary Figure 1F**). These findings were visually corroborated in the UMAP projection (**Supplementary Figure 1A**). To further characterize the melanoma TME and the features of melanoma cells, we performed a series of enrichment analyses. A word cloud analysis highlighted localization and the NDUFS gene family as most prominently associated with melanoma cells (**Supplementary Figure 1G**). GO-BP analysis revealed the top five enriched pathways in melanoma cells to be: oxidative phosphorylation, proton motive force–driven ATP synthesis, cellular respiration, aerobic respiration, and purine ribonucleoside triphosphate biosynthetic process (also purine nucleoside triphosphate biosynthetic process) (**Supplementary Figure 1H**). Upregulated genes in melanoma cells were primarily enriched in pathways such as chemical carcinogenesis–reactive oxygen species, prion disease, diabetic cardiomyopathy, fluid shear stress and atherosclerosis, Parkinson disease, and Huntington disease. In contrast, downregulated genes were associated with antigen processing and presentation, herpes simplex virus 1 infection, allograft rejection, phagosome, and type I diabetes mellitus (**Supplementary Figure 1I**). Differential expression analysis identified ITGAV, COX6C, and BFAR as the top differentially expressed genes in melanoma cells (**Supplementary Figure 1J**). GSEA further demonstrated upregulation of genes involved in cellular pigmentation, melanin biosynthetic process, and pigment metabolic process, alongside downregulation of genes related to lymphocyte differentiation, cellular response to tumor necrosis factor, and adaptive immune response (**Supplementary Figure 1K**).

### Single-cell characterization of melanoma cell subpopulations

**Figure 1:**
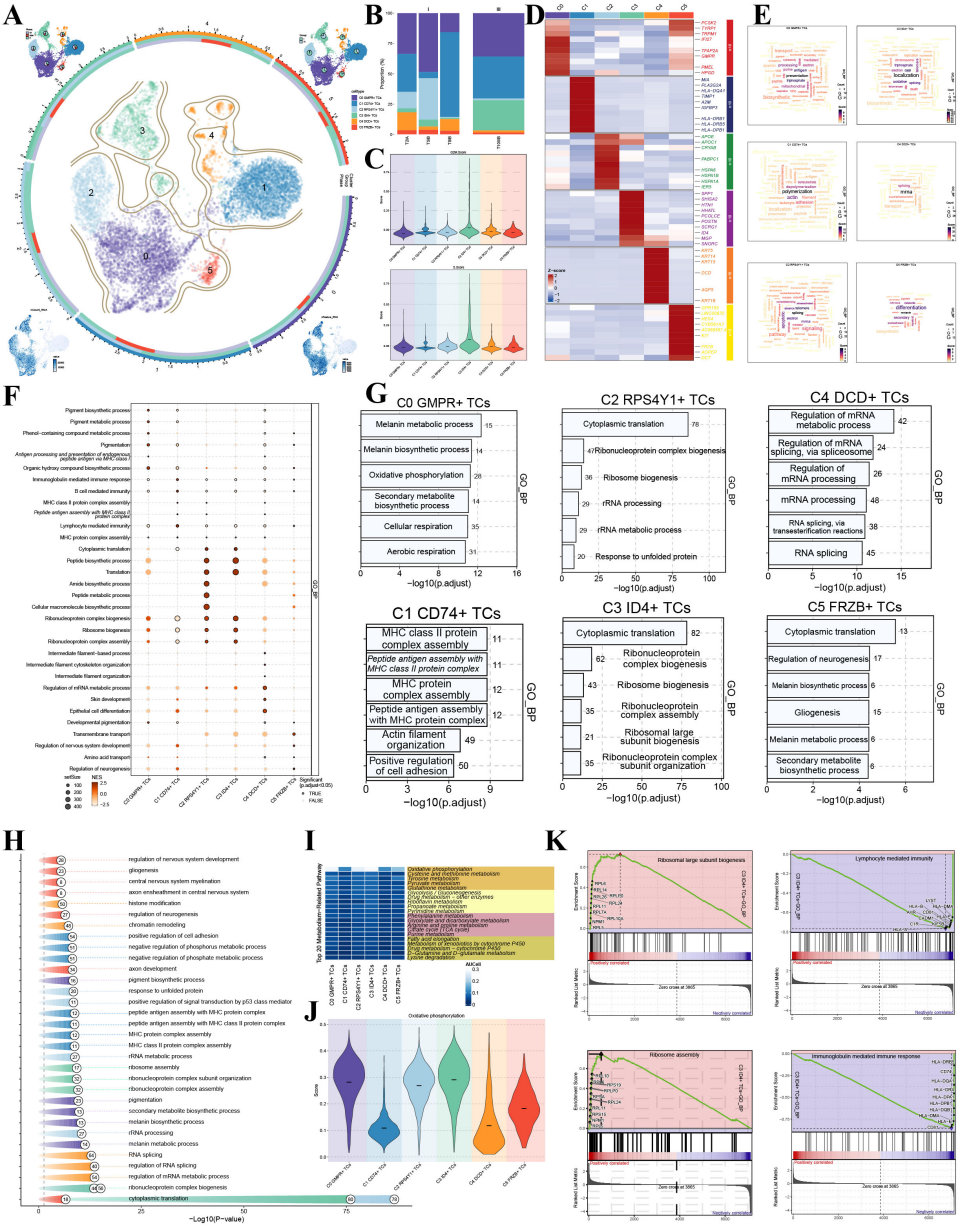
Heterogeneity of malignant melanoma tumor cells. **(A)** UMAP visualization of six tumor cell subpopulations annotated by specific marker genes. Top left: cell cycle distribution; Top right: tumor stage distribution; Bottom left: nCount_RNA distribution; Bottom right: nFeature_RNA distribution. **(B)** Proportion analysis of tumor cells stratified by tumor stage and data source. **(C)** Violin plots compared G2/M and S phase scores across different subpopulations. **(D)** Heatmap displayed differentially expressed genes among tumor cell subpopulations. **(E)** Word clouds illustrated highly associated biological processes for each subpopulation. **(F–H)** GO enrichment analysis of differentially expressed genes in tumor cells. **(I)** Metabolic pathways enriched in up-regulated and down-regulated genes across subpopulations. **(J)** Oxidative phosphorylation pathway activity scores for each subpopulation. **(K)** GO-GSEA analysis showed three significantly enriched terms for up-regulated genes and three for down-regulated genes in C3 ID4^+^ TCs.

Based on differentially expressed genes, we annotated and classified 9,335 tumor cells into six distinct subpopulations, designated according to their most significantly upregulated markers: C0 GMPR^+^ TCs (3,366 cells), C1 CD74^+^ TCs (2,895 cells), C2 RPS4Y1^+^ TCs (1,324 cells), C3 ID4^+^ TCs (1,048 cells), C4 DCD^+^ TCs (452 cells), C5 FRZB^+^ TCs (250 cells). Among these, C3 ID4^+^ TCs exhibited the highest proportion of cells in the G2/M and S phases and were predominantly derived from stage III tumors. C2 RPS4Y1^+^ TCs also showed a relatively high percentage of cells in G2/M and S phases but were almost exclusively derived from stage I tumors (**Figure 1A**). UMAP visualization revealed that C0 GMPR^+^ TCs, C2 RPS4Y1^+^ TCs, and C3 ID4^+^ TCs displayed markedly higher nCount_RNA and nFeature_RNA values compared to other subpopulations (**Figure 1A**). While C3 ID4^+^ TCs were scarcely present across stage I samples, their proportion increased substantially in stage III tumors, suggesting a strong association with disease progression (**Figure 1B**). Violin plots further confirmed that C3 ID4^+^ TCs had the highest G2/M and S phase scores among all subpopulations (**Figure 1C**). A heatmap of subpopulation-specific marker genes identified SPP1, SHISA2, HTN1, HHATL, PCOLCE, POSTN, SCRG1, ID4, MGP, and SNORC as distinctive markers for C3 ID4^+^ TCs (**Figure 1D**). To functionally characterize these subpopulations, we performed enrichment analyses. Word clouds illustrated the most enriched biological terms for each subpopulation: C0 GMPR^+^ TCs were most associated with presentation; C1 CD74^+^ TCs with polymerization; C2 RPS4Y1^+^ TCs with splicing; C3 ID4^+^ TCs with localization; C4 DCD^+^ TCs with mRNA; and C5 FRZB^+^ TCs with melanin (**Figure 1E**). GO-BP analysis revealed that C3 ID4^+^ TCs were most significantly enriched in cytoplasmic translation, followed by ribonucleoprotein complex biogenesis, ribosome biogenesis, ribonucleoprotein complex assembly, ribosomal large subunit biogenesis, and ribonucleoprotein complex subunit organization (**Figures 1F–H**). Metabolic pathway assessment based on AUCell values indicated strong enrichment of oxidative phosphorylation in C0 GMPR^+^ TCs, C2 RPS4Y1^+^ TCs, and C3 ID4^+^ TCs (**Figure 1I**). Subsequent scoring of oxidative phosphorylation activity confirmed that C3 ID4^+^ TCs exhibited the highest activity, followed by C0 GMPR^+^ TCs and C2 RPS4Y1^+^ TCs (**Figure 1J**). GSEA further highlighted upregulation of ribosomal large subunit biogenesis and ribosome assembly, alongside downregulation of lymphocyte mediated immunity and immunoglobulin mediated immune response in C3 ID4^+^ TCs (**Figure 1K**). In addition to the canonical malignant markers of this subpopulation, C3 ID4^+^ TCs also showed expression of EV-associated genes, including ANXA1 and SDCBP, suggesting a potential role in vesicle-related intercellular communication at the transcriptomic level. However, these findings should be interpreted as indirect molecular evidence rather than direct proof of enhanced EV secretion.

### Developmental trajectory and differentiation potential of melanoma cell subpopulations

**Figure 2:**
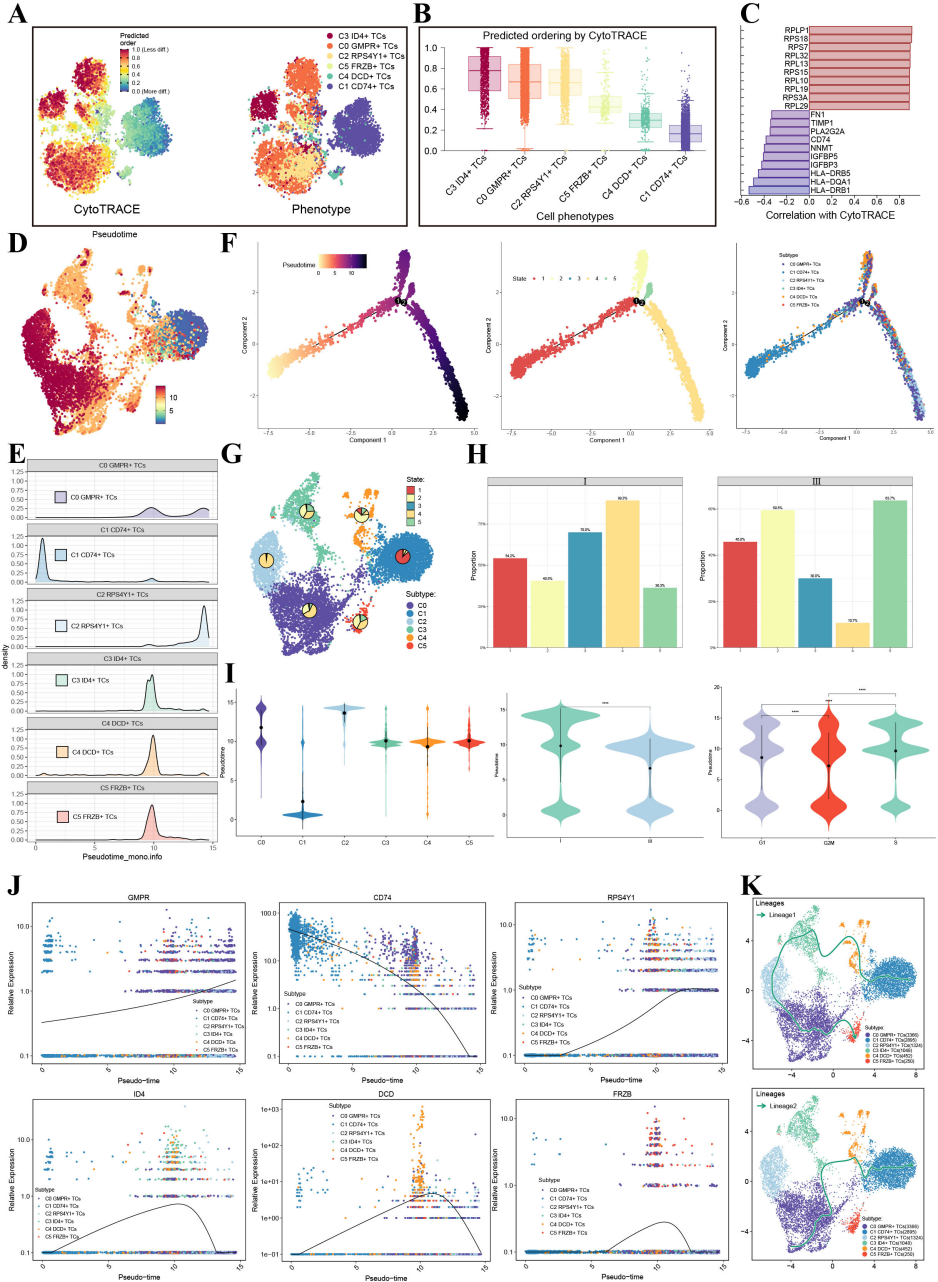
Developmental potential and trajectory of tumor cells. **(A, B)** CytoTRACE analysis of tumor cell subpopulations. **(C)** Genes associated with CytoTRACE analysis. **(D)** UMAP visualization of pseudotemporal ordering constructed by Monocle2. **(E)** Ridge plot displayed CytoTRACE scores across tumor cell subpopulations. **(F)** Monocle2 pseudotime trajectory visualization, divided into states 1-5, with subpopulation distribution along the trajectory. **(G)** UMAP showed proportional distribution of the five states across tumor subpopulations. **(H)** Percentage distribution of the five states across different tumor stages. **(I)** Violin plots showed pseudotime distribution across tumor subpopulations, tumor stages, and cell cycle phases. **(J)** Expression patterns of marker genes along the pseudotime axis for each subpopulation. **(K)** Two developmental trajectories (Lineage 1 and Lineage 2) constructed by Slingshot among tumor subpopulations.

To investigate the differentiation potential and developmental trajectories of melanoma cell subpopulations, we performed CytoTRACE analysis. UMAP visualization and box plots of CytoTRACE scores revealed that C3 ID4^+^ TCs exhibited the highest scores, indicating their relatively undifferentiated state and highest stem-like potential among all subpopulations, suggesting they may represent the earliest population along the differentiation continuum (**Figures 2A, B**). The genes utilized by CytoTRACE and their corresponding weights are displayed in **Figure 2C**. We next applied Monocle2 and Slingshot to reconstruct developmental trajectories and pseudotemporal ordering. Monocle2-based pseudotime reconstruction visualized on UMAP (**Figure 2D**) and corroborated by ridge plots (**Figure 2E**) demonstrated the distribution of all tumor subpopulations along the pseudotime axis. The inferred trajectory originated from the left (start) and progressed toward the right (end). Projection of subpopulations onto this trajectory showed that C1 CD74^+^ TCs were predominantly located near the start, while C2 RPS4Y1^+^ TCs and C3 ID4^+^ TCs clustered toward the terminal end. Based on branching points 1 and 2, the trajectory was partitioned into five distinct states (State 1–5, **Figure 2F**), where State 1 represented the initial phase of pseudotime, State 2 constituted the terminal branch after the first branch point, State 3 comprised the intermediate segment between branch points 1 and 2, and States 4 and 5 represented the two terminal branches following branch point 2. These pseudotime states should be interpreted as progression-associated transcriptional states rather than definitive terminal cell fates. Analysis of state distribution across subpopulations showed that C3 ID4^+^ TCs were predominantly enriched in States 2 and 4 at comparable proportions, whereas C0 GMPR^+^ TCs were most abundant in State 4 (**Figure 2G**). The preferential localization of C3 ID4+ TCs within States 2 and 4 suggests that these cells do not occupy a single terminal fate, but rather a pair of high-plasticity progression-associated states. Given their high CytoTRACE scores, proliferative activity, and enrichment for markers such as ID4, SPP1, and POSTN, State 2 may reflect a transitionally activated branch with stem-like and proliferative features, whereas State 4 may represent a more progression-committed branch linked to matrix remodeling and invasive behavior. Because state-specific differential expression analysis was not separately performed in the present study, these functional annotations should be considered provisional. Comparative analysis between tumor stages revealed that stage III samples exhibited a significant reduction in State 4 and a marked increase in State 5 compared to stage I tumors (**Figure 2H**). Violin plots further detailed pseudotime values across subpopulations, tumor stages, and cell cycle phases (**Figure 2I**). To visualize gene expression dynamics along pseudotime, we plotted expression trends of key marker genes, which showed that ID4—a specific marker for C3 ID4^+^ TCs—displayed a pronounced expression peak toward the end of pseudotime (**Figure 2J**). Complementary trajectory analysis using Slingshot reconstructed two distinct lineages: Lineage 1 followed the order C1 CD74^+^ TCs → C4 DCD^+^ TCs → C3 ID4^+^ TCs → C2 RPS4Y1^+^ TCs → C0 GMPR^+^ TCs, while Lineage 2 progressed as C0 GMPR^+^ TCs → C3 ID4^+^ TCs → C4 DCD^+^ TCs → C1 CD74^+^ TCs (**Figure 2K**). Together, these analyses provide a comprehensive view of the developmental hierarchy and differentiation dynamics within melanoma cell subpopulations.

### Cellular crosstalk between C3 ID4^+^ TCs and C0 GMPR^+^ TCs

**Figure 3:**
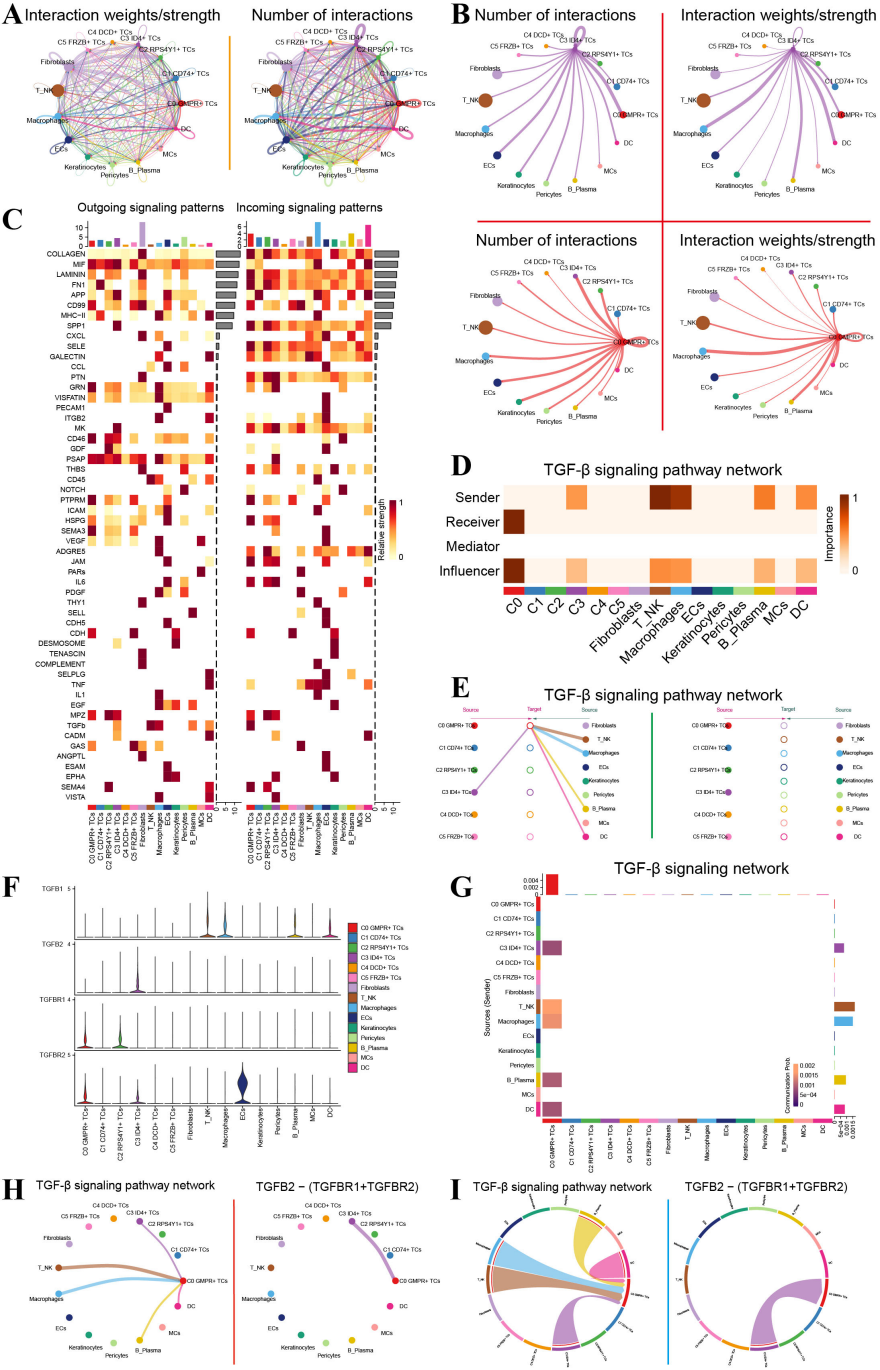
C3 ID4^+^ TCs communicate with C0 GMPR^+^ TCs through the TGFB2-(TGFBR1+TGFBR2) signaling axis. **(A)** Global overview of interaction number and interaction strength among tumor-cell subpopulations and microenvironmental cell types. **(B)** Focused view of interaction number and interaction strength centered on the C3 ID4+ TC and C0 GMPR+ TC axis. **(C)** Expression patterns of ligand- and receptor-related genes across all cell populations. **(D)** Importance scores of all cell types as signal senders, receivers, mediators, and influencers within the TGF-β signaling pathway. **(E)** Hierarchical network diagram of TGF-β-mediated intercellular communication, with different cell subpopulations designated as sources or targets. Arrow direction indicates signal flow, while line thickness represents communication strength. **(F)** Violin plots displayed expression levels of individual ligand and receptor components in the TGF-β pathway across all cell types. **(G)** Interaction network and communication strength mediated through TGF-β signaling. **(H)** Network plot of the TGF-β signaling pathway and the specific TGFB2-(TGFBR1+TGFBR2) interaction axis. **(I)** Circular plots illustrated global TGF-β signaling and the specific TGFB2-(TGFBR1+TGFBR2) communication axis.

CellChat was used to characterize intercellular communication across all cell types. **Figure 3A** summarizes the global communication landscape by showing the overall number and strength of interactions among major cell populations. **Figure 3B** highlights the principal signaling axis centered on C3 ID4^+^ TCs and C0 GMPR^+^ TCs. Signals from C3 ID4^+^ TCs to C0 GMPR^+^ TCs were particularly prominent in both number and strength. Conversely, C0 GMPR^+^ TCs received the highest number of incoming signals from C3 ID4^+^ TCs, while the strongest incoming signals were derived from macrophages. Heatmaps depicting outgoing and incoming signaling patterns revealed expression levels of ligand-receptor pairs across all cells (**Figure 3C**). Specifically, within outgoing signaling patterns, C3 ID4^+^ TCs showed elevated expression of TGF-β ligands, whereas in incoming signaling patterns, C0 GMPR^+^ TCs exhibited high expression of TGF-β-related receptors (**Figure 3C**). We further dissected the TGF-β signaling pathway given its relevance. In the TGF-β signaling network, C3 ID4^+^ TCs demonstrated high importance as signal senders, while C0 GMPR^+^ TCs displayed even greater importance as signal receivers (**Figure 3D**). Hierarchical visualization of TGF-β-mediated interactions confirmed that C3 ID4^+^ TCs act as primary sources communicating with C0 GMPR^+^ TCs (**Figure 3E**). Analysis of TGF-β pathway components showed that C0 GMPR^+^ TCs highly express both TGFBR1 and TGFBR2; C2 RPS4Y1^+^ TCs express TGFBR1; C3 ID4^+^ TCs express TGFB2 and TGFBR2; T/NK cells express TGFB1; macrophages express TGFB1; and endothelial cells (ECs) express TGFBR2 (**Figure 3F**). These findings were further validated by the specific signaling axis from C3 ID4^+^ TCs to C0 GMPR^+^ TCs via the TGF-β pathway (**Figure 3G**).

Collectively, these findings support an inferred TGFB2-(TGFBR1+TGFBR2) communication axis between C3 ID4^+^ TCs and C0 GMPR^+^ TCs (**Figures 3H, I**). Nevertheless, the downstream transcriptional consequences of this pathway in recipient cells, including canonical SMAD signaling and TGF-β-responsive transcriptional programs, could not be directly established in the present study and therefore require further validation.

### Transcriptional regulation in key tumor subpopulations

**Figure 4:**
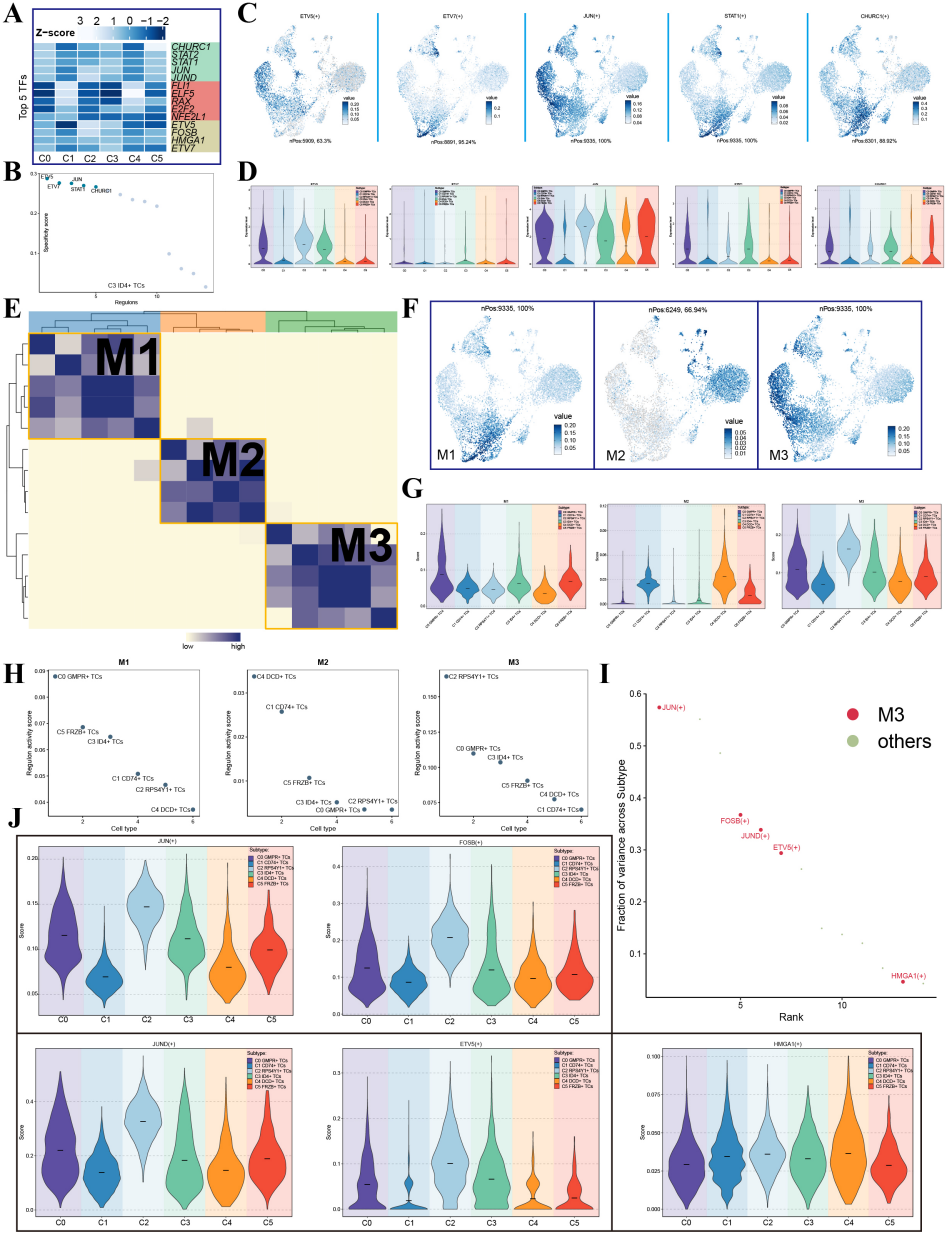
Transcription factor activity profile of C3 ID4^+^ TCs. **(A)** Heatmap displayed the top 5 TFs, transcription factors with highest transcriptional activity across tumor cell subpopulations. **(B)** The five transcription factors exhibiting the highest specificity scores in C3 ID4^+^ TCs. **(C)** UMAP visualization showed expression patterns of the top 5 TFs from C3 ID4^+^ TCs across all tumor cells. **(D)** Violin plots compared expression levels of the top 5 TFs from C3 ID4^+^ TCs among different tumor cell subpopulations. **(E)** Classification of transcription factors into three regulatory modules (M1, M2, M3) based on the CSI matrix. **(F)** Distribution of regulatory modules across tumor cell populations visualized on the original UMAP projection. **(G)** Violin plots compared regulatory module scores across tumor subpopulations. **(H)** Regulon activity scores of tumor cell subpopulations within the three regulatory modules. **(I)** Ranking of transcription factors in the M3 module based on their fraction of variance across subtypes. **(J)** Violin plots displayed activity scores of transcription factors JUN, FOSB, JUND, ETV5, and HMGA1 across tumor cell subpopulations.

We visualized the top five transcription factors (TFs) for each tumor subpopulation using a heatmap (**Figure 4A**). Among these, the top TFs identified for C3 ID4^+^ TCs were ETV5, ETV7, JUN, STAT1, and CHURC1. We further ranked all TFs in C3 ID4^+^ TCs based on their specificity scores, which confirmed the results shown in the heatmap (**Figure 4B**). UMAP visualization showed that these five TFs were enriched in C2 RPS4Y1^+^ TCs and C3 ID4^+^ TCs, with subtype-specific differences (**Figure 4C**). Violin plots were then used to compare expression levels of these TFs across tumor subpopulations (**Figure 4D**). ETV5 and JUN showed their highest expression in C2 RPS4Y1^+^ TCs and second-highest expression in C3 ID4^+^ TCs, whereas ETV7, STAT1, and CHURC1 displayed peak expression in C3 ID4^+^ TCs. In addition, JUN, STAT1, and CHURC1 were also notably expressed in C0 GMPR^+^ TCs. Based on the transcriptional regulatory activity matrix, we categorized the TFs of all tumor subpopulations into three regulatory modules: M1, M2, and M3 (**Figure 4E**). The distribution of these modules across subpopulations was visualized using UMAP (**Figure 4F**). Module M1 was primarily represented by C0 GMPR^+^ TCs, M2 mainly consisted of C1 CD74^+^ TCs and C4 DCD^+^ TCs, and M3 was dominated by C2 RPS4Y1^+^ TCs. Violin plots illustrated the activity levels of each module within the tumor subpopulations, revealing that M3 activity was strongest in C2 RPS4Y1^+^ TCs, followed by C0 GMPR^+^ TCs and C3 ID4^+^ TCs (**Figure 4G**). We further generated scatter plots to depict the relationship between tumor subpopulations and the regulatory modules based on their regulon activity scores (**Figure 4H**). In M1, C0 GMPR^+^ TCs ranked highest, while C4 DCD^+^ TCs scored the lowest. In M2, C4 DCD^+^ TCs ranked first, and C2 RPS4Y1^+^ TCs ranked last. In M3, C2 RPS4Y1^+^ TCs had the highest score, whereas C1 CD74^+^ TCs had the lowest. We then ranked the TFs in M3 according to their variance fraction across subpopulations, with the top TFs being JUN, FOSB, JUND, ETV5, and HMGA1 (**Figure 4I**). Violin plots were used to display the activity scores of these five TFs across tumor subpopulations (**Figure 4J**). JUN, FOSB, and JUND showed the highest activity in C2 RPS4Y1^+^ TCs, followed by C0 GMPR^+^ TCs and C3 ID4^+^ TCs. ETV5 was most active in C2 RPS4Y1^+^ TCs, with C3 ID4^+^ TCs ranking second. In contrast, HMGA1 activity was relatively uniform across all subpopulations.

### ETV5 depletion suppresses melanoma cell proliferation and migration while promoting apoptosis

**Figure 5:**
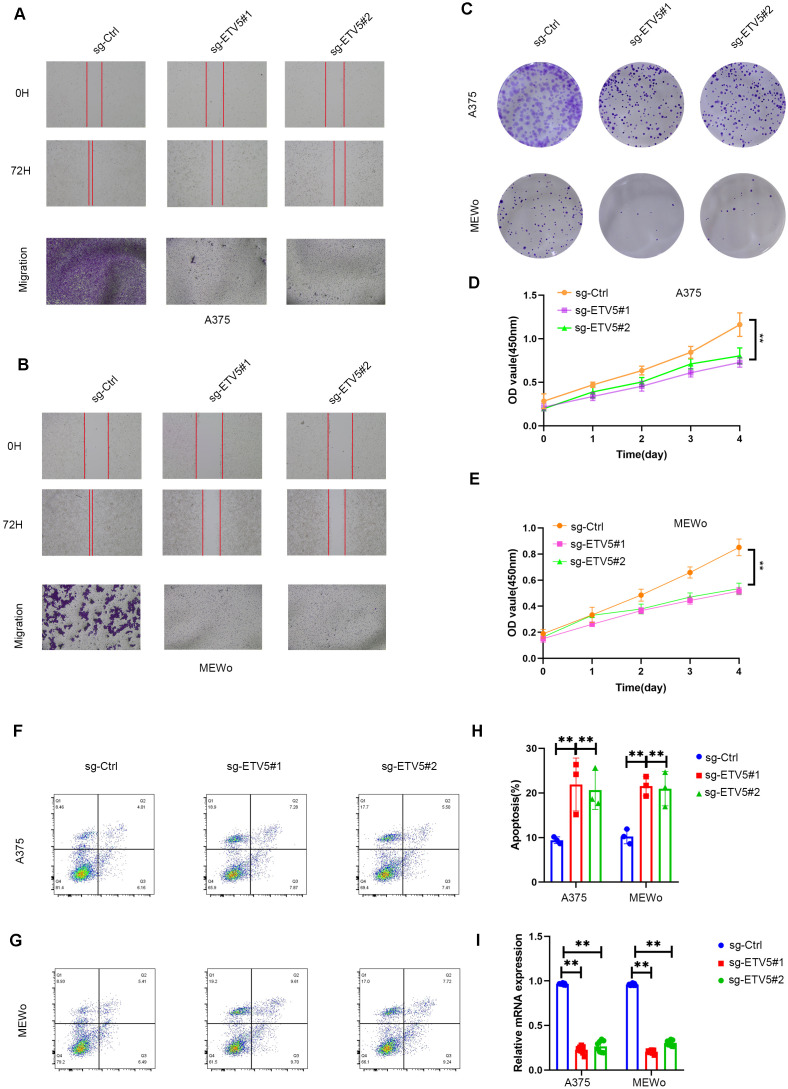
ETV5 knockdown suppresses melanoma cell migration and proliferation while promoting apoptosis. **(A, B)** Scratch wound healing and Transwell migration assays in A375 **(A)** and MEWo **(B)** cells transduced with sg-Ctrl or two independent sgRNAs targeting ETV5 (sg-ETV5#1 and sg-ETV5#2). Representative images at 0 h and 72 h are shown. **(C)** Colony formation assays demonstrated reduced clonogenic capacity upon ETV5 knockdown. **(D, E)** Cell proliferation assessed by CCK-8 assay in A375 **(D)** and MEWo **(E)** cells over 4 days. **(G)** Flow cytometry analysis of apoptosis in A375 **(F)** and MEWo **(G)** cells. **(H)** Quantification of apoptotic cell percentages. **(I)** RT-qPCR analysis confirming decreased ETV5 mRNA expression. Data are presented as mean ± SD. **p < 0.01; one-way ANOVA.

Based on our single‐cell transcription factor regulatory network analysis which identified ETV5 as a putative oncogenic driver associated with enhanced proliferative and migratory phenotypes in malignant clusters, we next performed functional assays to validate its biological role in melanoma cells. CRISPR-mediated knockdown of ETV5 (sg-ETV5#1 and sg-ETV5#2) in A375 and MEWo cells markedly impaired migratory capacity, as evidenced by slower wound closure in scratch assays compared with sg-Ctrl cells (**Figures 5A, B**). Consistently, Transwell migration assays showed a substantial reduction in the number of migrated cells following ETV5 silencing in both cell lines, confirming impaired motility.

ETV5 depletion also significantly restrained melanoma cell growth. Colony formation assays demonstrated a pronounced decrease in colony numbers in ETV5-knockdown groups (**Figure 5C**), and CCK-8 proliferation curves revealed a continuous suppression of cell viability over 4 days in A375 and MEWo cells (**Figures 5D, E**).

In contrast, ETV5 knockdown promoted cell apoptosis. Flow cytometry analysis showed increased early and late apoptotic cell fractions in sg-ETV5 cells compared with controls (**Figures 5F, G**), which was quantitatively confirmed (**Figure 5H**).

RT-qPCR further verified the effective silencing of ETV5 expression in both cell lines (**Figure 5I**).

Together, these findings functionally validate our single-cell analysis, supporting that ETV5 acts as a pro‐tumorigenic transcription factor in melanoma, promoting tumor cell migration and proliferation while suppressing programmed cell death. It should be noted that transcription factor transcript abundance and inferred regulon activity are not necessarily equivalent. SCENIC-based activity reflects downstream target engagement within a specific regulatory context rather than mRNA level alone; therefore, partial discordance between ETV5 expression and ETV5 activity across subpopulations is biologically plausible and may reflect differences in cofactors, chromatin accessibility, or target-gene activation.

## Discussion

Malignant melanoma is a highly heterogeneous and aggressive form of skin cancer. Although the application of immune checkpoint inhibitors and targeted therapies has significantly improved survival outcomes in some patients with advanced disease, challenges such as heterogeneous treatment responses, drug resistance, and immune-related adverse events remain substantial (54–56). TME plays a critical role in melanoma initiation, progression, and immune evasion, and the heterogeneity in its cellular composition and molecular characteristics is a major contributor to interpatient variability in treatment response (57–59). In recent years, EVs have emerged as key mediators of intercellular communication within the TME, capable of transferring proteins, lipids, and nucleic acids to modulate immune responses and therapy resistance. However, the specific tumor subpopulations that are major sources of these EVs, and the molecular mechanisms through which they exert their effects, remain poorly defined.

In this study, we performed an integrated analysis of single-cell RNA sequencing data from 10 Stage I and Stage III malignant melanoma tissues to systematically characterize the cellular composition of the TME, transcriptional features of tumor cell subpopulations, differentiation potential, and intercellular communication networks. We identified multiple tumor cell subpopulations with distinct molecular phenotypes and functional states, among which the C3 ID4^+^ TCs subpopulation exhibited prominent features associated with tumor progression, cell cycle activity, oxidative phosphorylation metabolism, and stemness. Further pseudotime trajectory analysis, differentiation potential assessment, and cell–cell communication studies revealed the potential driver role of C3 ID4^+^ TCs in melanoma progression. We also highlighted the crosstalk between C3 ID4^+^ TCs and C0 GMPR^+^ TCs mediated by the TGF-β signaling pathway, and deciphered key transcriptional regulatory networks underlying these subpopulations. By providing a systematic single-cell perspective, this study elucidates the heterogeneous nature of the melanoma TME and its functional implications in tumor progression, offering a novel theoretical foundation for the development of future targeted and immunotherapeutic strategies.

Initially, we performed unbiased clustering analysis on all cells derived from Stage I and Stage III melanoma tissues, successfully mapping the cellular landscape of the tumor microenvironment and identifying 10 major cell clusters. These included melanoma cells, fibroblasts, T/NK cells, macrophages, endothelial cells, keratinocytes, pericytes, B/plasma cells, mast cells, and dendritic cells. This comprehensive classification establishes a foundation for understanding the complex composition of the melanoma TME. Notably, we observed a significant increase in the proportion of melanoma cells in Stage III samples, along with a higher percentage of these cells in the G2/M and S phases of the cell cycle. This pattern visually reflects the trend of enhanced proliferative activity and the increasing dominance of tumor cells within the TME as the disease progresses. Analysis of stemness characteristics revealed elevated stemness scores in endothelial cells, pericytes, and melanoma cells, consistent with the potential roles of these cell types in tumor angiogenesis, structural support, and tumor initiation (60–63). Importantly, the high stemness features observed in melanoma cells themselves suggest the possible existence of subpopulations with self-renewal and differentiation potential, providing a rationale for further sub-classification of malignant-prone subsets within the tumor cell population. Enrichment analysis of the broader melanoma cell population revealed significant metabolic reprogramming features, including enrichment in oxidative phosphorylation and ATP synthesis pathways, alongside downregulation of pathways related to antigen presentation and adaptive immune responses. These overall characteristics indicate that melanoma cells, as a whole, adapt to survival and progression pressures by enhancing energy metabolism and suppressing immune recognition. This insight directs our subsequent focus on intra-tumoral heterogeneity and the identification of specific subpopulations that drive this malignant phenotype (64).

We subsequently identified six distinct tumor cell subpopulations, among which the C3 ID4^+^ TCs subpopulation was significantly enriched in Stage III tumor samples and exhibited the highest proportion of cells in G2/M and S phases, indicating elevated proliferative activity. We employed CytoTRACE to assess differentiation potential across subpopulations and found that C3 ID4^+^ TCs displayed the highest CytoTRACE score, suggesting a relatively undifferentiated state, high differentiation potential, and likely increased malignancy. In the pseudotime trajectory reconstructed by Monocle2, C1 CD74^+^ TCs were predominantly located at the starting segment, while C2 RPS4Y1^+^ TCs and C3 ID4^+^ TCs clustered toward the terminal end, potentially reflecting differences in differentiation states and developmental ordering among subpopulations. In the Slingshot-derived Lineage 1 and Lineage 2 trajectories, C3 ID4^+^ TCs—characterized by high CytoTRACE scores—were positioned at relatively early pseudotime points. Further analysis revealed that C3 ID4^+^ TCs highly express genes such as ID4, SPP1, and POSTN. ID4, an inhibitor of DNA-binding proteins, is involved in the pathogenesis of multiple cancers and has been shown not only to promote tumor growth, invasion, and metastasis but also to maintain self-renewal in embryonic stem cells (65, 66). SPP1 (osteopontin) can bind and activate multiple downstream signaling pathways, thereby stimulating tumor growth and invasion while limiting the anti-tumor functions of immune cells. For instance, integrin αvβ3 binding to SPP1 promotes cell migration via FAK, ERK1/2, and NF-κB signaling, and facilitates tumor progression while reducing apoptosis through the PI3K/Akt/mTOR and JAK2/STAT3 pathways (67–69). POSTN (periostin), a secreted extracellular matrix protein, participates in cancer stem cell maintenance and metastatic progression, and high POSTN expression is associated with poor prognosis (70, 71). These findings collectively suggest that the C3 ID4^+^ TCs subpopulation may exhibit a more aggressive phenotype and serve as a key driver of melanoma progression. To further characterize the C3 ID4^+^ TCs subpopulation, we performed enrichment analysis across all tumor subpopulations. We found that C3 ID4^+^ TCs displayed prominent metabolic reprogramming, with significant enrichment in oxidative phosphorylation (OXPHOS). As a central process in cellular energy reprogramming, upregulation of OXPHOS helps meet the energetic and biosynthetic demands of rapidly proliferating and metastasizing tumor cells (72–75). Moreover, OXPHOS can promote genetic instability, proliferation, and immune evasion through the generation of reactive oxygen species (ROS) (76, 77). In melanoma, upregulation of OXPHOS has been linked to BRAF inhibitor resistance (78). Our study further validated the high activity of OXPHOS in C3 ID4^+^ TCs using AUCell analysis and metabolic pathway scoring, indicating that this subpopulation may enhance its survival advantage and malignant phenotype through metabolic reprogramming. In addition to OXPHOS, the C3 ID4^+^ TCs subpopulation was significantly enriched in cytoplasmic translation-related pathways, such as ribosome biogenesis and ribonucleoprotein complex assembly. Upregulation of ribosome biogenesis is a hallmark of rapidly proliferating tumor cells, supporting cell cycle progression and tumor growth by enhancing protein synthesis (79, 80). Our GO-BP and GSEA analyses further confirmed strong enrichment of ribosome large subunit biogenesis and ribosome assembly pathways in C3 ID4^+^ TCs, suggesting that this subpopulation may promote tumor cell proliferation and survival through upregulation of cytoplasmic translation. We also observed downregulation of multiple immune-related pathways in C3 ID4^+^ TCs, including immunoglobulin production, lymphocyte-mediated immunity, and immunoglobulin-mediated immune response. Such downregulation may contribute to T cell dysfunction and immune evasion within the tumor microenvironment. Furthermore, C3 ID4^+^ TCs showed reduced expression of genes involved in immune cell infiltration and activation, such as MHC class I and II molecules, which may represent a key mechanism for evading immune surveillance (81). Recent studies have shown that EVs derived from OXPHOS-high or metabolically stressed tumor cells can transfer mitochondrial components (e.g., mitochondrial DNA, respiratory chain proteins) and metabolites to recipient cells, thereby conferring drug resistance and enhancing survival (82). We propose that the C3 ID4^+^ TCs subpopulation, through its distinct metabolic and translational profile, may generate EVs with a unique molecular signature that promotes resistance to targeted therapy and immunotherapy. These EVs could potentially transfer resistance-conferring molecules to more differentiated tumor cells or to immune cells, thereby acting as a propagating mechanism for therapy failure.

Furthermore, our CellChat analysis revealed prominent TGF-β signaling-mediated crosstalk between the C3 ID4^+^ TCs and C0 GMPR^+^ TCs subpopulations. The TGF-β signaling pathway plays a dual role in tumor progression, acting as a tumor suppressor in early stages while promoting metastasis and immune evasion in advanced disease (83–87). In this study, we found that C3 ID4^+^ TCs highly express TGFB2 and TGFBR2, whereas C0 GMPR^+^ TCs show elevated expression of TGFBR1 and TGFBR2, suggesting that TGF-β signaling may serve as an important regulatory axis between these two subpopulations. Transcriptional regulators play crucial roles in tumor initiation and progression. Beyond direct ligand-receptor interactions, a growing body of evidence suggests that TGF-β family members, along with other immunomodulatory and pro-metastatic factors, are frequently packaged into EVs and delivered to recipient cells within the TME (88, 89). Notably, C3 ID4^+^ TCs also highly express SPP1 and POSTN, both of which have been identified as EV cargo components in other cancers and are known to promote immune suppression, angiogenesis, and metastasis (90, 91). This raises the compelling hypothesis that C3 ID4^+^ TCs may function as a major source of immunosuppressive EVs within the melanoma TME. These EVs could serve as durable, long-range carriers of TGFB2, SPP1, POSTN, and potentially other oncogenic molecules, thereby amplifying their influence beyond immediate cellular neighbors and contributing to a systemic immunosuppressive state.

Our single-cell transcriptomic analysis identified ETV5, ETV7, JUN, STAT1, and CHURC1 as the top five transcription factors with the highest expression in the C3 ID4^+^ TCs subpopulation. ETV5, a member of the ETS transcription factor family, has been implicated in cell proliferation, migration, and apoptosis resistance across multiple cancer types (92–95). In hepatocellular carcinoma, tumor cell-derived ETV5 activates the expression of PD-L1 and S100A9. Elevated S100A9 recruits polymorphonuclear myeloid-derived suppressor cells (MDSCs) and further upregulates ETV5 expression in both tumor cells and MDSCs through the ERK/NF-κB pathway. Additionally, ETV5 enhances PD-L1 expression in MDSCs, thereby strengthening tumor immune evasion capabilities (96). Based on the transcriptional regulatory activity matrix, we categorized the transcription factors across all tumor subpopulations into three regulatory modules (M1–M3). Within the M3 module, which showed the highest regulon activity score for C3 ID4^+^ TCs, ETV5 was ranked among the top transcription factors in terms of activity—consistent with the expression patterns described above. This positions ETV5 not only as a driver of an aggressive cellular phenotype but also as a potential regulator of EV production or cargo selection in this critical subpopulation. Future studies are warranted to investigate whether ETV5 directly regulates genes involved in EV biogenesis or influences the packaging of specific miRNAs or proteins into EVs released by melanoma cells. Given that piRNAs can accumulate in exosomes and have been implicated in cancer proliferation, metastasis, chemoresistance, stemness, and biomarker development (97), future studies should investigate whether EVs released by C3 ID4^+^ TCs contain functionally relevant piRNA cargosADDIN.

To validate the functional role of ETV5 in melanoma, we employed CRISPR/Cas9-mediated knockdown of ETV5 expression. Subsequent functional assays demonstrated that ETV5 depletion significantly impaired melanoma cell migration, as evidenced by both wound healing and Transwell migration assays. Furthermore, colony formation assays revealed sustained suppression of proliferative capacity in ETV5-deficient melanoma cells. Flow cytometry analysis additionally indicated an increased proportion of apoptotic cells upon ETV5 knockdown. These experimental results align with our single-cell analysis findings and collectively support the tumor-promoting function of ETV5 in melanoma pathogenesis. Given our proposed link between C3 ID4^+^ TCs and EV-mediated signaling, an intriguing future direction would be to examine whether ETV5 knockdown alters the quantity, composition, or functional properties of EVs released by these cells. If ETV5 is indeed a regulator of an “EV-release program” in aggressive melanoma cells, then targeting ETV5 could have the dual benefit of inhibiting intrinsic tumor cell malignancy and disrupting a key intercellular communication vector that sustains the immunosuppressive TME.

While this study has uncovered the potential role of the C3 ID4^+^ TCs subpopulation in melanoma progression through single-cell RNA sequencing and functional experiments, several limitations should be acknowledged. First, the analysis relied on scRNA-seq data from public databases with a limited sample size, which may affect the generalizability of the findings. Future multi-center studies with larger cohorts are warranted to validate these observations. Although we identified ETV5 as a key transcription factor, its precise regulatory network and downstream target genes, especially those related to EV biology, remain incompletely characterized. Finally, while this work primarily focused on the characteristics of tumor cell subpopulations and proposes an EV-mediated communication model, direct experimental validation is required. Future studies should isolate EVs from sorted C3 ID4^+^ TCs (and control subpopulations) using established methods (e.g., ultracentrifugation, size-exclusion chromatography) and perform proteomic, lipidomic, and RNA-seq analyses to define their cargo. Functional assays, such as incubating these EVs with immune cells (T cells, macrophages) or other tumor cells, are crucial to confirm their role in suppressing immune function, promoting metastasis, or conferring drug resistance.

In summary, this study identified the C3 ID4^+^ TCs subpopulation as a highly malignant subset in melanoma through single-cell RNA sequencing analysis. Integrated enrichment analysis, CellChat communication mapping, and transcriptional regulator profiling revealed its distinct characteristics in oxidative phosphorylation, cytoplasmic translation, immune evasion, and intercellular signaling. Functional validation confirmed the critical role of the transcription factor ETV5 within this subpopulation. Based on the concurrent enrichment of oxidative phosphorylation pathways and the expression of EV-associated factors in C3 ID4^+^ TCs, together with cell-cell communication results, we propose an extended mechanistic model: ETV5 transcriptional activity is upregulated in C3 ID4^+^ TCs, driving a pro-metastatic and immunosuppressive gene program. This program includes the production of soluble factors (e.g., TGFB2, SPP1, POSTN) that may also be selectively loaded into EVs. These EVs then disseminate through the TME and potentially systemically, reprogramming recipient cells (including immune cells and other tumor cells) to foster immune evasion, enhance OXPHOS, and promote therapy resistance. Despite the need for direct validation of EV involvement, this work provides new insights into melanoma heterogeneity and progression mechanisms and explicitly links a high-risk cellular subset to the emerging paradigm of EV-mediated tumor-stroma communication. Our findings suggest that targeting ETV5, TGF-β signaling, or the EV release pathway itself may represent a promising multi-pronged therapeutic strategy for malignant melanoma, particularly in tumors enriched with the C3 ID4^+^ TCs subpopulation. Furthermore, EVs derived from this subpopulation or specific EV cargos (e.g., TGFB2, POSTN) could be developed as novel prognostic or predictive biomarkers for melanoma aggressiveness and immunotherapy resistance.

## Data Availability

The single-cell RNA sequencing dataset analyzed in this study is publicly available in the GEO database under accession number GSE277165. The original contributions generated in this study are included in the article/Supplementary Material. Further inquiries can be directed to the corresponding authors.

## Supplementary material

Supplementary Figure 1 Cellular heterogeneity in malignant melanoma tissues. **(A)** UMAP visualization of all cell types following dimensionality reduction and clustering. Top left: Cell Stemness AUC scores; Top right: nCount_RNA values; Bottom left: S phase scores; Bottom right: G2/M phase scores. **(B)** Bubble plot displayed the top 5 marker genes for each cell population. **(C)** Proportional distribution of cell types across different tumor stages. **(D)** Percentage distribution of cell types across cell cycle phases. **(E)** Heatmap showed the Ro/e (Ratio of observed to expected) values for all cell types across different tumor stages. **(F)** Box plots compared Cell Stemness AUC, nCount_RNA, S phase, and G2/M phase scores across all cell populations. **(G)** Word cloud illustrated cellular behaviors and genes highly associated with tumor cells. **(H)** GO-BP enrichment analysis based on differentially expressed genes in melanoma cells. **(I)** Gene set enrichment profiles in tumor cells. **(J)** GO-BP enrichment analysis for all cell types based on differentially expressed genes. **(K)** Six representative enrichment terms associated with melanoma cells from GSEA analysis.

## References

[1] Wang J Zhao F Zhang Q Sun Z Xiahou Z Wang C . Unveiling the NEFH+ Malignant cell subtype: Insights from single-cell RNA sequencing in prostate cancer progression and tumor microenvironment interactions. Front Immunol. (2024) 15:1517679. doi: 10.3389/fimmu.2024.1517679. PMID: 39759507 PMC11695424

[2] Tong Z Mang G Wang D Cui J Yang Q Zhang M . Single-cell RNA sequencing maps immune cell heterogeneity in mice with allogeneic cardiac transplantation. Cardiovasc Innov Appl. (2023) 8 (1):988. doi: 10.15212/cvia.2023.0023

[3] Wang M Cao Y Ren C Wang K Wang Y Wu X . PTRF confers melanoma-acquired drug resistance through the upregulation of EGFR. Cell Prolif. (2025) 59:e70086. doi: 10.1111/cpr.70086. PMID: 40745979 PMC12877943

[4] Hou M Zhao Z Li S Zhang Z Li X Zhang Y . Single-cell analysis unveils cell subtypes of acral melanoma cells at the early and late differentiation stages. J Cancer. (2025) 16:898–916. doi: 10.7150/jca.102045. PMID: 39781353 PMC11705046

[5] Sun R Wei S Yu Y Wang Z Yao T Zhang Y . Prognostic value and immune infiltration of a tumor microenvironment-related PTPN6 in metastatic melanoma. Cancer Cell Int. (2024) 24:435. doi: 10.1186/s12935-024-03625-6. PMID: 39732710 PMC11682626

[6] Zheng R Zhuang Z Zhao C Zhao Z Yang X Zhou Y . Chinese admission warning strategy for predicting the hospital discharge outcome in patients with traumatic brain injury. J Clin Med. (2022) 11 (4):974. doi: 10.3390/jcm11040974. PMID: 35207247 PMC8880692

[7] Zhu X Che X Yang R Bao B Chu G . Global, regional, and national burden of cancer in the elderly population, 1990–2021: Analysis of data from the Global Burden of Disease Study 2021. Med Res. (2025) 1:mdr2-70031. doi: 10.1002/mdr2.70031. PMID: 41889077

[8] Lai G Xie B Zhang C Zhong X Deng J Li K . Comprehensive analysis of immune subtype characterization on identification of potential cells and drugs to predict response to immune checkpoint inhibitors for hepatocellular carcinoma. Genes Dis. (2024) 12:101471. doi: 10.1016/j.gendis.2024.101471. PMID: 40092490 PMC11907441

[9] Lai G Li K Deng J Liu H Xie B Zhong X . Identification and validation of a gene signature for lower-grade gliomas based on pyroptosis-related genes to predict survival and response to immune checkpoint inhibitors. J Healthc Eng. (2022) 2022:8704127. doi: 10.1155/2022/8704127. PMID: 35535221 PMC9078805

[10] Zhao Z Cai H Zhao Z Wang X Nie W Zhao F . Cancer-associated fibroblast-derived GDF15 induces oxidative stress and neutrophil infiltration in head and neck squamous cell carcinoma through the PI3K/AKT/STAT3 axis cascade. Res (Wash D C). (2025) 8:901. doi: 10.34133/research.0901. PMID: 41035818 PMC12480759

[11] Shi M Wang J Huang X Liu L Liu L Zhou N . Targeting TMEM16A/ANO1 inhibits the progression of BRAF mutant (V600E) melanoma through the MEK/ERK and AKT signaling pathways. Genes Dis. (2024) 11:101153. doi: 10.1016/j.gendis.2023.101153. PMID: 39070550 PMC11278794

[12] Guo L Wang C Qiu X Pu X Chang P . Colorectal cancer immune infiltrates: Significance in patient prognosis and immunotherapeutic efficacy. Front Immunol. (2020) 11:1052. doi: 10.3389/fimmu.2020.01052. PMID: 32547556 PMC7270196

[13] Atanasova VS Riedl A Strobl M Flandorfer J Unterleuthner D Weindorfer C . Selective eradication of colon cancer cells harboring PI3K and/or MAPK pathway mutations in 3D culture by combined PI3K/AKT/mTOR pathway and MEK inhibition. Int J Mol Sci. (2023) 24 (2):1668. doi: 10.3390/ijms24021668. PMID: 36675180 PMC9863259

[14] Zhao J Li D Xie S Deng X Wen X Li J . Nomogram for predicting prognosis of patients with metastatic melanoma after immunotherapy: A Chinese population-based analysis. Front Immunol. (2022) 13:1083840. doi: 10.3389/fimmu.2022.1083840. PMID: 36618343 PMC9815596

[15] Lai G Zhong X Liu H Deng J Li K Xie B . A novel m7G-related genes-based signature with prognostic value and predictive ability to select patients responsive to personalized treatment strategies in bladder cancer. Cancers (Basel). (2022) 14:5346. doi: 10.3390/cancers14215346. PMID: 36358764 PMC9656096

[16] Lai G Liu H Deng J Li K Zhang C Zhong X . The characteristics of tumor microenvironment predict survival and response to immunotherapy in adrenocortical carcinomas. Cells. (2023) 12:755. doi: 10.3390/cells12050755. PMID: 36899891 PMC10000893

[17] Coelho JQ Ramos MJ Ranchor R Pichel R Guerra L Miranda H . What's new about the tumor microenvironment of urothelial carcinoma? Clin Transl Oncol. (2024) 26:1549–60. doi: 10.1007/s12094-024-03384-w. PMID: 38332225

[18] Lin L Zou J Pei S Huang W Zhang Y Zhao Z . Germinal center B-cell subgroups in the tumor microenvironment cannot be overlooked: Their involvement in prognosis, immunotherapy response, and treatment resistance in head and neck squamous carcinoma. Heliyon. (2024) 10:e37726. doi: 10.1016/j.heliyon.2024.e37726. PMID: 39391510 PMC11466559

[19] Li S Yao J Zhang S Zhou X Zhao X Di N . Prognostic value of tumor-microenvironment-associated genes in ovarian cancer. Bio Integration. (2023) 4 (3):84–96. doi: 10.15212/bioi-2022-0008

[20] Gao Z Jiang A Li Z Zhu L Mou W Shen W . Heterogeneity of intratumoral microbiota within the tumor microenvironment and relationship to tumor development. Med Res. (2025) 1:32–61. doi: 10.1002/mdr2.70006. PMID: 41889077

[21] Ning K Peng Y Jiang Y Li Z Luo X Lin L . Sex differences in renal cell carcinoma: A single-cell analysis reveals exhausted CD8(+) T-cells highly infiltrated in males. Biol Sex Differ. (2023) 14:58. doi: 10.1186/s13293-023-00540-9. PMID: 37715192 PMC10503187

[22] Hinshaw DC Shevde LA . The tumor microenvironment innately modulates cancer progression. Cancer Res. (2019) 79:4557–66. doi: 10.1158/0008-5472.CAN-18-3962. PMID: 31350295 PMC6744958

[23] Wang Z Wu X . Study and analysis of antitumor resistance mechanism of PD1/PD-L1 immune checkpoint blocker. Cancer Med. (2020) 9:8086–121. doi: 10.1002/cam4.3410. PMID: 32875727 PMC7643687

[24] Takabe P Siiskonen H Ronka A Kainulainen K Pasonen-Seppanen S . The impact of hyaluronan on tumor progression in cutaneous melanoma. Front Oncol. (2021) 11:811434. doi: 10.3389/fonc.2021.811434. PMID: 35127523 PMC8813769

[25] Xue F Liu YK Chen XY Chen SS Yu XR Li HW . Targeting cGAS-STING: Modulating the immune landscape of hepatic diseases. Front Immunol. (2025) 16:1498323. doi: 10.3389/fimmu.2025.1498323. PMID: 40098962 PMC11911377

[26] Jin MZ Jin WL . The updated landscape of tumor microenvironment and drug repurposing. Signal Transduct Target Ther. (2020) 5:166. doi: 10.1038/s41392-020-00280-x. PMID: 32843638 PMC7447642

[27] Zhao Z Cai H Nie W Wang X Zhao Z Zhao F . Ectopic expression of GDF15 in cancer-associated fibroblasts enhances melanoma immunosuppression via the GFRAL/RET cascade. J Immunother Cancer. (2025) 13 (6):e011036. doi: 10.1136/jitc-2024-011036. PMID: 40555562 PMC12198796

[28] Romano V Belviso I Venuta A Ruocco MR Masone S Aliotta F . Influence of tumor microenvironment and fibroblast population plasticity on melanoma growth, therapy resistance and immunoescape. Int J Mol Sci. (2021) 22 (10):5283. doi: 10.3390/ijms22105283. PMID: 34067929 PMC8157224

[29] Yin Y Liu B Cao Y Yao S Liu Y Jin G . Colorectal cancer-derived small extracellular vesicles promote tumor immune evasion by upregulating PD-L1 expression in tumor-associated macrophages. Adv Sci (Weinh). (2022) 9:2102620. doi: 10.1002/advs.202102620. PMID: 35356153 PMC8948581

[30] Pryczynicz A Cepowicz D Zareba K Gryko M Holody-Zareba J Kedra B . Dysfunctions in the mature dendritic cells are associated with the presence of metastases of colorectal cancer in the surrounding lymph nodes. Gastroenterol Res Pract. (2016) 2016:2405437. doi: 10.1155/2016/2405437. PMID: 26839537 PMC4709662

[31] Kraja FP Jurisic VB Hromic-Jahjefendic A Rossopoulou N Katsila T Mirjacic Martinovic K . Tumor-infiltrating lymphocytes in cancer immunotherapy: From chemotactic recruitment to translational modeling. Front Immunol. (2025) 16:1601773. doi: 10.3389/fimmu.2025.1601773. PMID: 40475782 PMC12137109

[32] Liu ZL Liu JH Staiculescu D Chen J . Combination of molecularly targeted therapies and immune checkpoint inhibitors in the new era of unresectable hepatocellular carcinoma treatment. Ther Adv Med Oncol. (2021) 13:17588359211018026. doi: 10.1177/17588359211018026. PMID: 34104226 PMC8150670

[33] Li K Huang J Tan Y Sun J Zhou M . Single-cell and bulk transcriptome analysis reveals tumor cell heterogeneity and underlying molecular program in uveal melanoma. J Transl Med. (2024) 22:1020. doi: 10.1186/s12967-024-05831-2. PMID: 39533334 PMC11555829

[34] Liu X Xie J Xiao Y . SLC3A2 as a key anoikis-related gene for prognosis and tumor microenvironment remodeling in melanoma. Discov Oncol. (2025) 16:1306. doi: 10.1007/s12672-025-03125-7. PMID: 40643718 PMC12254447

[35] Clancy JW D'Souza-Schorey C . Tumor-derived extracellular vesicles: Multifunctional entities in the tumor microenvironment. Annu Rev Pathol. (2023) 18:205–29. doi: 10.1146/annurev-pathmechdis-031521-022116. PMID: 36202098 PMC10410237

[36] Xie F Zhou X Fang M Li H Su P Tu Y . Extracellular vesicles in cancer immune microenvironment and cancer immunotherapy. Adv Sci (Weinh). (2019) 6:1901779. doi: 10.1002/advs.201901779. PMID: 31871860 PMC6918121

[37] Xie J Zheng Z Tuo L Deng X Tang H Peng C . Recent advances in exosome-based immunotherapy applied to cancer. Front Immunol. (2023) 14:1296857. doi: 10.3389/fimmu.2023.1296857. PMID: 38022585 PMC10662326

[38] Leary N Walser S He Y Cousin N Pereira P Gallo A . Melanoma-derived extracellular vesicles mediate lymphatic remodelling and impair tumour immunity in draining lymph nodes. J Extracell Vesicles. (2022) 11:e12197. doi: 10.1002/jev2.12197. PMID: 35188342 PMC8859913

[39] Fleming V Hu X Weller C Weber R Groth C Riester Z . Melanoma extracellular vesicles generate immunosuppressive myeloid cells by upregulating PD-L1 via TLR4 signaling. Cancer Res. (2019) 79:4715–28. doi: 10.1158/0008-5472.CAN-19-0053. PMID: 31337655

[40] Xie S Cai Y Chen D Xiang Y Cai W Mao J . Single-cell transcriptome analysis reveals heterogeneity and convergence of the tumor microenvironment in colorectal cancer. Front Immunol. (2022) 13:1003419. doi: 10.3389/fimmu.2022.1003419. PMID: 36685571 PMC9845924

[41] Hong R Tong Y Tang H Zeng T Liu R . eMCI: An explainable multimodal correlation integration model for unveiling spatial transcriptomics and intercellular signaling. Res (Wash D C). (2024) 7:522. doi: 10.34133/research.0522. PMID: 39494219 PMC11528068

[42] Chen Q Guo X Wang H Sun S Jiang H Zhang P . Plasma-free blood as a potential alternative to whole blood for transcriptomic analysis. Phenomics. (2024) 4:109–24. doi: 10.1007/s43657-023-00121-1. PMID: 38884056 PMC11169349

[43] Luo S Le Wang Xiao Y Cao C Liu Q Zhou Y . Single-cell RNA-sequencing integration analysis revealed immune cell heterogeneity in five human autoimmune diseases. Bio Integration. (2023) 4 (4):145–59. doi: 10.15212/bioi-2023-0012

[44] Du H Li S Lu J Tang L Jiang X He X . Single-cell RNA-seq and bulk-seq identify RAB17 as a potential regulator of angiogenesis by human dermal microvascular endothelial cells in diabetic foot ulcers. Burns Trauma. (2023) 11:tkad020. doi: 10.1093/burnst/tkad020. PMID: 37605780 PMC10440157

[45] Cheng XC Tong WZ Rui W Feng Z Shuai H Zhe W . Single-cell sequencing technology in skin wound healing. Burns Trauma. (2024) 12:tkae043. doi: 10.1093/burnst/tkae043. PMID: 39445224 PMC11497848

[46] Tang W Sun G Ji GW Feng T Zhang Q Cao H . Single-cell RNA-sequencing atlas reveals an FABP1-dependent immunosuppressive environment in hepatocellular carcinoma. J Immunother Cancer. (2023) 11 (11):e007030. doi: 10.1136/jitc-2023-007030. PMID: 38007237 PMC10679975

[47] Ren S Pan R Wang Z . Multi-omics and single cell sequencing analyses reveal associations of mitophagy-related genes predicting clinical prognosis and immune infiltration characteristics in osteosarcoma. Mol Biotechnol. (2025) 67:3583–98. doi: 10.1007/s12033-024-01280-w. PMID: 39264525

[48] Cha J Kim DH Kim G Cho JW Sung E Baek S . Single-cell analysis reveals cellular and molecular factors counteracting HPV-positive oropharyngeal cancer immunotherapy outcomes. J Immunother Cancer. (2024) 12 (6):e008667. doi: 10.1136/jitc-2023-008667. PMID: 38857913 PMC11168198

[49] Sun Y You Y Wu Q Hu R Dai K . Senescence-targeted microRNA/organoid composite hydrogel repair cartilage defect and prevention joint degeneration via improved chondrocyte homeostasis. Bioact Mater. (2024) 39:427–42. doi: 10.1016/j.bioactmat.2024.05.036. PMID: 38855061 PMC11157121

[50] Li H Bian Y Xiahou Z Zhao Z Zhao F Zhang Q . The cellular signaling crosstalk between memory B cells and tumor cells in nasopharyngeal carcinoma cannot be overlooked: Their involvement in tumor progression and treatment strategy is significant. J Cancer. (2025) 16:288–314. doi: 10.7150/jca.101420. PMID: 39744570 PMC11660138

[51] Ouyang Y Hong Y Mai C Yang H Wu Z Gao X . Transcriptome analysis reveals therapeutic potential of NAMPT in protecting against abdominal aortic aneurysm in human and mouse. Bioact Mater. (2024) 34:17–36. doi: 10.1016/j.bioactmat.2023.11.020. PMID: 38173843 PMC10761368

[52] Li CZ Hu TY . Nanotechnology powered CRISPR-Cas systems for point of care diagnosis and therapeutic. Res (Wash D C). (2022) 2022:9810237. doi: 10.34133/2022/9810237. PMID: 36157513 PMC9484831

[53] Dong X Gou Y Guo M Zhong J Li A Hao A . A simplified noncryogenic strategy to transport mesenchymal stem cells: Potential applications in cell therapy and regenerative medicine. Genes Dis. (2024) 11:101073. doi: 10.1016/j.gendis.2023.07.002. PMID: 38274386 PMC10808911

[54] Verginadis II Avgousti H Monslow J Skoufos G Chinga F Kim K . A stromal integrated stress response activates perivascular cancer-associated fibroblasts to drive angiogenesis and tumour progression. Nat Cell Biol. (2022) 24:940–53. doi: 10.1038/s41556-022-00918-8. PMID: 35654839 PMC9203279

[55] Yang Z Jia Y Wang S Zhang Y Fan W Wang X . Retinoblastoma-binding protein 5 regulates H3K4 methylation modification to inhibit the proliferation of melanoma cells by inactivating the Wnt/beta-catenin and epithelial-mesenchymal transition pathways. J Oncol. (2023) 2023:5093941. doi: 10.1155/2023/5093941. PMID: 36866240 PMC9974310

[56] Ge Q Zhao Z Li X Yang F Zhang M Hao Z . Deciphering the suppressive immune microenvironment of prostate cancer based on CD4+ regulatory T cells: Implications for prognosis and therapy prediction. Clin Transl Med. (2024) 14:e1552. doi: 10.1002/ctm2.1552. PMID: 38239097 PMC10797244

[57] Niu W Yang Y Teng Y Zhang N Li X Qin Y . Pan-cancer analysis of PGAM1 and its experimental validation in uveal melanoma progression. J Cancer. (2024) 15:2074–94. doi: 10.7150/jca.93398. PMID: 38434965 PMC10905406

[58] Diazzi S Tartare-Deckert S Deckert M . The mechanical phenotypic plasticity of melanoma cell: an emerging driver of therapy cross-resistance. Oncogenesis. (2023) 12:7. doi: 10.1038/s41389-023-00452-8. PMID: 36774337 PMC9922263

[59] Zhao Z Ding Y Tran LJ Chai G Lin L . Innovative breakthroughs facilitated by single-cell multi-omics: manipulating natural killer cell functionality correlates with a novel subcategory of melanoma cells. Front Immunol. (2023) 14:1196892. doi: 10.3389/fimmu.2023.1196892. PMID: 37435067 PMC10332463

[60] Wen Y Ma L Liu Y Xiong H Shi D . Decoding the enigmatic role of T-cadherin in tumor angiogenesis. Front Immunol. (2025) 16:1564130. doi: 10.3389/fimmu.2025.1564130. PMID: 40230838 PMC11994602

[61] Inukai M Yokoi A Ishizuka Y Hashimura M Matsumoto T Oguri Y . A functional role of S100A4/non-muscle myosin IIA axis for pro-tumorigenic vascular functions in glioblastoma. Cell Commun Signal. (2022) 20:46. doi: 10.1186/s12964-022-00848-w. PMID: 35392912 PMC8991692

[62] Ye J Gao X Huang X Huang S Zeng D Luo W . Integrating single-cell and spatial transcriptomics to uncover and elucidate GP73-mediated pro-angiogenic regulatory networks in hepatocellular carcinoma. Res (Wash D C). (2024) 7:387. doi: 10.34133/research.0387. PMID: 38939041 PMC11208919

[63] Zhao Z Dong Y Zhao Z Xiahou Z Sun C . Single-cell atlas of endothelial cells in atherosclerosis: identifying C1 CXCL12+ ECs as key proliferative drivers for immunological precision therapeutics in atherosclerosis. Front Immunol. (2025) 16:1569988. doi: 10.3389/fimmu.2025.1569988. PMID: 40421026 PMC12104226

[64] Ni D Zhou H Wang P Xu F Li C . Visualizing macrophage phenotypes and polarization in diseases: From biomarkers to molecular probes. Phenomics. (2023) 3:613–38. doi: 10.1007/s43657-023-00129-7. PMID: 38223685 PMC10781933

[65] Chen HJ Yu Y Sun YX Huang CZ Li JY Liu F . Id4 suppresses the growth and invasion of colorectal cancer HCT116 cells through CK18-related inhibition of AKT and EMT signaling. J Oncol. (2021) 2021:6660486. doi: 10.1155/2021/6660486. PMID: 33936204 PMC8060092

[66] Zhang Y Zhang LX Liu XQ Zhao FY Ge C Chen TY . Id4 promotes cell proliferation in hepatocellular carcinoma. Chin J Cancer. (2017) 36:19. doi: 10.1186/s40880-017-0186-7. PMID: 28143562 PMC5286768

[67] Bie T Zhang X . Higher expression of SPP1 predicts poorer survival outcomes in head and neck cancer. J Immunol Res. (2021) 2021:8569575. doi: 10.1155/2021/8569575. PMID: 34977258 PMC8718292

[68] Ruan S Rody WJJ Patel SS Hammadi LI Martin ML de Faria LP . Receptor activator of nuclear factor-kappa B is enriched in CD9-positive extracellular vesicles released by osteoclasts. Extracellular Vesicles Circulating Nucleic Acids. (2023) 4:518–29. doi: 10.20517/evcna.2023.38. PMID: 37936884 PMC10629932

[69] Huang DF Zhang WJ Chen J Jiao ZG Wang XL Rao DY . Hepatocellular carcinoma cell-derived small extracellular vesicle-associated CD147 serves as a diagnostic marker and promotes endothelial cell angiogenesis via the PI3K/Akt pathway. Extracellular Vesicles Circulating Nucleic Acids. (2023) 4:532–47. doi: 10.20517/evcna.2023.30. PMID: 40357132 PMC12066417

[70] Dorafshan S Razmi M Safaei S Gentilin E Madjd Z Ghods R . Periostin: biology and function in cancer. Cancer Cell Int. (2022) 22:315. doi: 10.1186/s12935-022-02714-8. PMID: 36224629 PMC9555118

[71] Xu C Wang Z Zhang L Feng Y Lv J Wu Z . Periostin promotes the proliferation and metastasis of osteosarcoma by increasing cell survival and activates the PI3K/Akt pathway. Cancer Cell Int. (2022) 22:34. doi: 10.1186/s12935-021-02441-6. PMID: 35057799 PMC8780812

[72] An Y Zhao F Jia H Meng S Zhang Z Li S . Inhibition of programmed cell death by melanoma cell subpopulations reveals mechanisms of melanoma metastasis and potential therapeutic targets. Discov Oncol. (2025) 16:62. doi: 10.1007/s12672-025-01789-9. PMID: 39832036 PMC11747064

[73] LeBleu VS O'Connell JT Gonzalez Herrera KN Wikman H Pantel K Haigis MC . PGC-1alpha mediates mitochondrial biogenesis and oxidative phosphorylation in cancer cells to promote metastasis. Nat Cell Biol. (2014) 16:992–1003. doi: 10.1038/ncb3039. PMID: 25241037 PMC4369153

[74] Ren L Meng L Gao J Lu M Guo C Li Y . PHB2 promotes colorectal cancer cell proliferation and tumorigenesis through NDUFS1-mediated oxidative phosphorylation. Cell Death Dis. (2023) 14:44. doi: 10.1038/s41419-023-05575-9. PMID: 36658121 PMC9852476

[75] Zhao Z Zhao Z Lin Z Fan L Xiahou Z Dong Y . Decoding multiple myeloma: single-cell insights into tumor heterogeneity, immune dynamics, and disease progression. Front Immunol. (2025) 16:1584350. doi: 10.3389/fimmu.2025.1584350. PMID: 40406148 PMC12095158

[76] Carretero-Fernandez M Cabrera-Serrano AJ Sanchez-Maldonado JM Ruiz-Duran L Jimenez-Romera F Garcia-Verdejo FJ . Autophagy and oxidative stress in solid tumors: mechanisms and therapeutic opportunities. Crit Rev Oncol Hematol. (2025) 212:104820. doi: 10.1016/j.critrevonc.2025.104820. PMID: 40580999

[77] Shen J Yang D Ding Y . Advances in promoting the efficacy of chimeric antigen receptor T cells in the treatment of hepatocellular carcinoma. Cancers (Basel). (2022) 14(20):5018. doi: 10.3390/cancers14205018. PMID: 36291802 PMC9599749

[78] Carpenter EL Chagani S Nelson D Cassidy PB Laws M Ganguli-Indra G . Mitochondrial complex I inhibitor deguelin induces metabolic reprogramming and sensitizes vemurafenib-resistant BRAF(V600E) mutation bearing metastatic melanoma cells. Mol Carcinog. (2019) 58:1680–90. doi: 10.1002/mc.23068. PMID: 31211467 PMC6692247

[79] Sun Y Li Y Zhang A Hu T Li M . Prognostic model identification of ribosome biogenesis-related genes in pancreatic cancer based on multiple machine learning analyses. Discov Oncol. (2025) 16:905. doi: 10.1007/s12672-025-02733-7. PMID: 40411705 PMC12103412

[80] Yang Y Zhang Y Ren J Feng K Li Z Huang T . Identification of colon immune cell marker genes using machine learning methods. Life (Basel). (2023) 13(9):1876. doi: 10.3390/life13091876. PMID: 37763280 PMC10532943

[81] Nasr S Li L Asad M Moridi M Wang M Zemp FJ . A computational pipeline for identifying gene targets and signalling pathways in cancer cells to improve lymphocyte infiltration and immune checkpoint therapy efficacy. EBioMedicine. (2024) 104:105167. doi: 10.1016/j.ebiom.2024.105167. PMID: 38805852 PMC11154126

[82] He X Zhong L Wang N Zhao B Wang Y Wu X . Gastric cancer actively remodels mechanical microenvironment to promote chemotherapy resistance via MSCs-mediated mitochondrial transfer. Adv Sci (Weinh). (2024) 11:e2404994. doi: 10.1002/advs.202404994. PMID: 39392399 PMC11653701

[83] Rodrigues-Junior DM Tsirigoti C Psatha K Kletsas D Aivaliotis M Heldin CH . TGF-beta induces cholesterol accumulation to regulate the secretion of tumor-derived extracellular vesicles. J Exp Clin Cancer Res. (2025) 44:42. doi: 10.1186/s13046-025-03291-0. PMID: 39910665 PMC11800471

[84] Gupta A Singh AK Kumar R Ganguly R Rana HK Pandey PK . Corilagin in cancer: a critical evaluation of anticancer activities and molecular mechanisms. Molecules. (2019) 24 (18):3399. doi: 10.3390/molecules24183399. PMID: 31546767 PMC6767293

[85] Qu H Zhao J Zuo X He H Wang X Li H . TGF-beta-mediated activation of fibroblasts in cervical cancer: implications for tumor microenvironment and prognosis. PeerJ. (2025) 13:e19072. doi: 10.7717/peerj.19072. PMID: 40124621 PMC11929507

[86] Zhao S Xie J Zhang Q Ni T Lin J Gao W . New anti-fibrotic strategies for keloids: insights from single-cell multi-omics. Cell Prolif. (2025) 58:e13818. doi: 10.1111/cpr.13818. PMID: 39902627 PMC12179555

[87] Thirugnanam K Rizvi F Jahangir A Homar P Shabnam F Palecek SP . SNRK facilitates cardiac repair associated with nonischemic fibrosis: regulating transforming growth factor-beta1 levels in atrial cardiomyocytes. Regener Med Rep. (2025) 2:45–52. doi: 10.4103/regenmed.regenmed-d-25-00009. PMID: 40584789 PMC12204380

[88] Rodrigues-Junior DM Tsirigoti C Psatha K Kletsas D Aivaliotis M Heldin CH . TGF-β induces cholesterol accumulation to regulate the secretion of tumor-derived extracellular vesicles. J Exp Clin Cancer Res. (2025) 44:42. doi: 10.1186/s13046-025-03291-0. PMID: 39910665 PMC11800471

[89] Teixeira AF Wang Y Iaria J Ten Dijke P Zhu HJ . Extracellular vesicles secreted by cancer-associated fibroblasts drive non-invasive cancer cell progression to metastasis via TGF-β signalling hyperactivation. J Extracell Vesicles. (2025) 14:e70055. doi: 10.1002/jev2.70055. PMID: 40091448 PMC11911544

[90] Bhadresha K Upadhyay V Brahmbhatt J Mughal MJ Jain N Rawal R . *In vitro* model of predicting metastatic ability using tumor derived extracellular vesicles; beyond seed soil hypothesis. Sci Rep. (2022) 12:20258. doi: 10.1038/s41598-022-24443-8. PMID: 36424413 PMC9691738

[91] Castellani G Buccarelli M D'Alessandris QG Ilari R Cappannini A Pedini F . Extracellular vesicles produced by irradiated endothelial or glioblastoma stem cells promote tumor growth and vascularization modulating tumor microenvironment. Cancer Cell Int. (2024) 24:72. doi: 10.1186/s12935-024-03253-0. PMID: 38347567 PMC10863174

[92] Su H Zhao L Fang T Han W Fan H . Identification of ETV5 as a prognostic marker related to epigenetic modification in pan-cancer and facilitates tumor progression in hepatocellular carcinoma. Sci Rep. (2024) 14:29695. doi: 10.1038/s41598-024-81642-1. PMID: 39614096 PMC11607464

[93] Zhang L Fu R Liu P Wang L Liang W Zou H . Biological and prognostic value of ETV5 in high-grade serous ovarian cancer. J Ovarian Res. (2021) 14:149. doi: 10.1186/s13048-021-00899-6. PMID: 34736492 PMC8570011

[94] De Bastiani MA Klamt F . Integrated transcriptomics reveals master regulators of lung adenocarcinoma and novel repositioning of drug candidates. Cancer Med. (2019) 8:6717–29. doi: 10.1002/cam4.2493. PMID: 31503425 PMC6825976

[95] Martinez VD Vucic EA Pikor LA Thu KL Hubaux R Lam WL . Frequent concerted genetic mechanisms disrupt multiple components of the NRF2 inhibitor KEAP1/CUL3/RBX1 E3-ubiquitin ligase complex in thyroid cancer. Mol Cancer. (2013) 12:124. doi: 10.1186/1476-4598-12-124. PMID: 24138990 PMC4016213

[96] Zhang Z Huang W Hu D Jiang J Zhang J Wu Z . E-twenty-six-specific sequence variant 5 (ETV5) facilitates hepatocellular carcinoma progression and metastasis through enhancing polymorphonuclear myeloid-derived suppressor cell (PMN-MDSC)-mediated immunosuppression. Gut. (2025) 74:1137–49. doi: 10.1136/gutjnl-2024-333944. PMID: 40015948

[97] Deng X Liao T Xie J Kang D He Y Sun Y . The burgeoning importance of PIWI-interacting RNAs in cancer progression. Sci China Life Sci. (2024) 67:653–62. doi: 10.1007/s11427-023-2491-7. PMID: 38198029

