# Supplementary figures and images for: Single-cell landscape of melanoma reveals ETV5-driven C3 ID4+ tumor subpopulation with extracellular vesicle-associated immunosuppressive and pro-metastatic potential
Source: Front Immunol. 2026 Apr 16;17:1795778. doi: 10.3389/fimmu.2026.1795778 (PMC13128620; doi:10.3389/fimmu.2026.1795778)

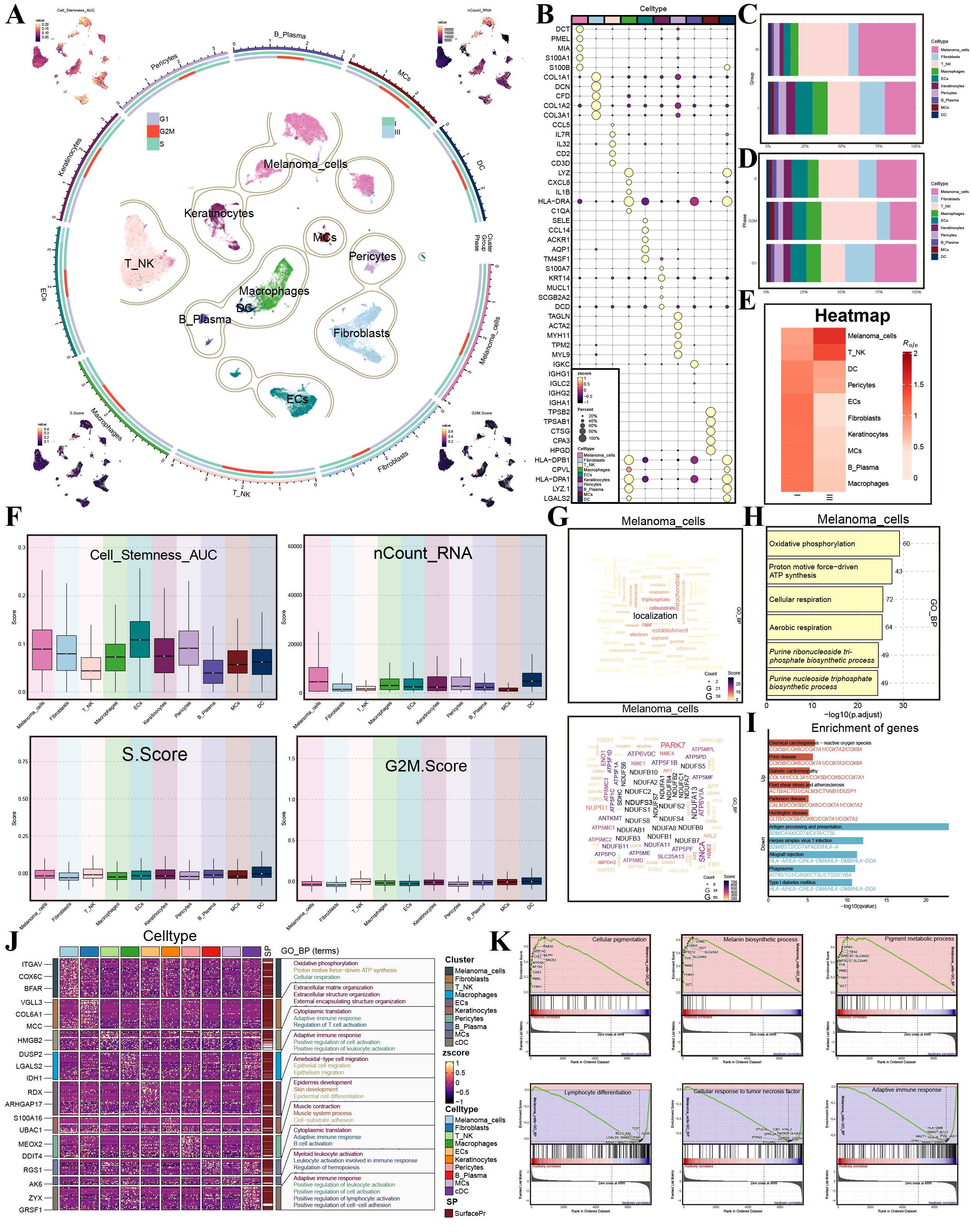

Supplement: Supplementary Figure 1 — Cellular heterogeneity in malignant melanoma tissues. (A) UMAP visualization of all cell types following dimensionality reduction and clustering. Top left: Cell Stemness AUC scores; Top right: nCount_RNA values; Bottom left: S phase scores; Bottom right: G2/M phase scores. (B) Bubble plot displayed the top 5 marker genes for each cell population. (C) Proportional distribution of cell types across different tumor stages. (D) Percentage distribution of cell types across cell cycle phases. (E) Heatmap showed the Ro/e (Ratio of observed to expected) values for all cell types across different tumor stages. (F) Box plots compared Cell Stemness AUC, nCount_RNA, S phase, and G2/M phase scores across all cell populations. (G) Word cloud illustrated cellular behaviors and genes highly associated with tumor cells. (H) GO-BP enrichment analysis based on differentially expressed genes in melanoma cells. (I) Gene set enrichment profiles in tumor cells. (J) GO-BP enrichment analysis for all cell types based on differentially expressed genes. (K) Six representative enrichment terms associated with melanoma cells from GSEA analysis. [file Image1.jpeg]
